# Redox-Active Polyindole-Modified Electrode for Electrochemical Uric Acid Determination

**DOI:** 10.1021/acsomega.6c08422

**Published:** 2026-09-02

**Authors:** Alam Zeb, Salma Bilal, Bushra Begum, Anwar ul Haq Ali Shah, Philipp Röse

**Affiliations:** † National Centre of Excellence in Physical Chemistry, 54736 University of Peshawar, Peshawar, Khyber Pakhtunkhwa 25120, Pakistan; ‡ Department of Chemistry, Women University, Mardan 23200, Pakistan; § Institute of Chemical Sciences, University of Peshawar, Peshawar, Khyber Pakhtunkhwa 25120, Pakistan; ∥ Institute for Applied Materials − Electrochemical Technologies, Karlsruhe Institute of Technology, Adenauerring 20b, Karlsruhe 76131, Germany

## Abstract

Uric acid (UA) is an important clinical biomarker whose reliable determination in complex biological media is often hampered by coexisting electroactive interferents. Intrinsically conducting polymers are versatile materials for electrochemical sensing, yet polyindole (PIn) has remained largely unexplored for this purpose. Here, we report a PIn-modified glassy carbon electrode for the nonenzymatic determination of UA in neutral phosphate buffer. PIn was synthesized by chemical oxidative polymerization in the presence of sulfonate dopants and deposited as a thin film on glassy carbon; physicochemical characterization confirmed a doped, conjugated, and nanostructured coating with a high effective surface area. The electrode showed a well-defined UA oxidation signal and, by chronoamperometry, a linear response over 0.01–0.10 mM with a sensitivity of 4.53 μA cm^–2^ mM^–1^ and a limit of detection of 4.0 μM. Under the applied conditions, ascorbic acid, dopamine, glucose, and sodium chloride contributed only marginally to the response, and the sensor performed reliably in diluted human serum, with recoveries of 99.7–99.9% and relative standard deviations below 5%. These findings identify polyindole as a promising, still underexplored intrinsically conducting polymer for electrochemical biosensing.

## Graphical abstract

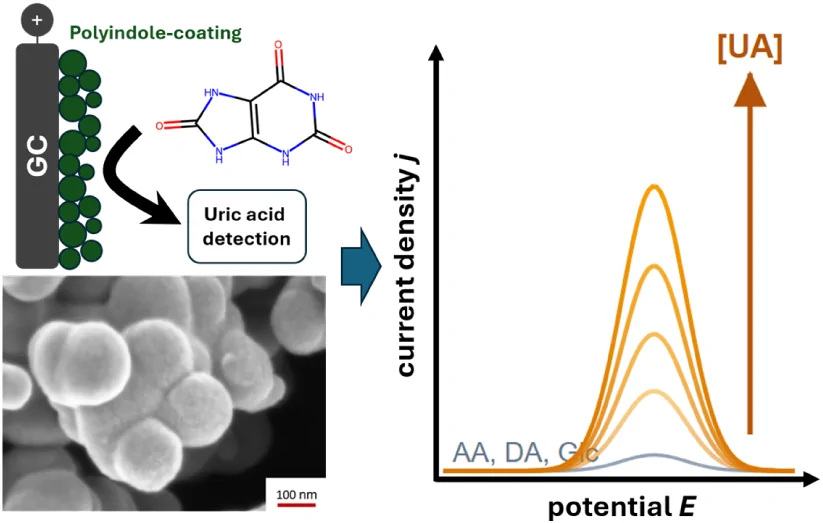

## 1 Introduction

Uric acid (UA) is the final product of purine metabolism in humans and is predominantly produced in the liver. In healthy adults, the physiological serum UA levels in males typically range from 3.5 to 7.2 mg/dL (0.21–0.43 mM), whereas in premenopausal females they range from 2.6 to 6.0 mg/dL (0.16–0.36 mM). These levels are maintained by a balance between dietary intake and endogenous production via nucleic acid catabolism and de novo synthesis.^1^ Beyond being a metabolic end product, UA also acts as an important antioxidant and may protect the central nervous system by mitigating oxidative damage and neuronal cell death, thereby influencing the development and progression of Parkinson’s disease (PD). However, elevated UA levels have also been reported to impair vascular endothelial integrity.^2^ By initiating inflammatory processes required for tissue repair, scavenging reactive oxygen species, and activating progenitor endothelial cells, UA may play a key role in tissue healing.^3^ UA concentrations are also key indicators in diagnosis, prognosis, and treatment of various renal disorders.^4^ Abnormal UA levels are associated with a range of diseases, including gout, hyperuricemia, Lesch–Nyhan syndrome, and kidney and cardiovascular abnormalities, while low UA has been linked to multiple sclerosis, Parkinson’s disease, and Alzheimer’s disease. Therefore, accurate monitoring of UA in body fluids is of high clinical relevance and may enable earlier diagnosis and improved disease treatment.

Numerous analytical methods have been developed for the determination of UA, including colorimetry,^5^ liquid chromatography,^6^ enzymatic methods,^7^ chemiluminescence,^8^ fluorescence spectroscopy. However, these conventional techniques often rely on expensive instrumentation, involve lengthy and intricate procedures.^9^ In contrast, electrochemical methods provide a powerful alternative for UA detection due to their high sensitivity and selectivity, low instrumentation cost, facile integration with portable readout electronics, and rapid response times.^10–12^ Owing to electrode downsizing and miniaturization, electrochemical sensing platforms can be compact, lightweight, and user-friendly, which is particularly attractive for point-of-care applications. Among the various electroanalytical approaches, voltammetric (current measured as a function of potential) and amperometric (current measured as a function of time) techniques are the most widely employed for UA sensing.^13^ Although UA is an electroactive species and can in principle be quantified by these methods, its determination in biological fluids is complicated by the presence of other electroactive biomolecules, such as glucose, ascorbic acid (AA), and dopamine (DA), which generate overlapping signals and thereby hinder selective UA detection.^14^ Consequently, these interferents must be carefully considered when designing electrochemical sensors for UA in physiological media. Improving the selectivity and sensitivity of UA determination can be achieved by modifying the electrode surface with suitable functional materials.

A broad range of materials has been used to modify sensor electrodes, in many cases to combine a large effective surface area, high electrical conductivity, and favorable mechanical and chemical stability.^15,16^ Among them, intrinsically conducting polymers (ICPs) constitute a particularly versatile class of functional materials, offering tunable chemical and physical properties, including adjustable molecular architecture, structural versatility, environmental stability, and pronounced sensitivity to their local chemical environment.^17–22^ Established ICPs such as polyaniline (PANI), polypyrrole (PPy), poly­(para-phenylene) (PPP), and poly­(3,4-ethylenedioxythiophene):poly­(styrenesulfonate) (PEDOT:PSS) have shown considerable potential for UA sensing.^23–29^ Their high electronic conductivity facilitates efficient charge transfer when the polymers are deposited on various electrode substrates by chemical or electrochemical methods. PPy and PPP are generally reported to be stable in both aqueous and nonaqueous media, whereas PANI provides distinct redox behavior with multiple oxidation states, allowing it to act as an electron-transfer mediator. These properties have established ICPs as key components in the development of electrochemical sensors and biosensors,^15,30^ and the field continues to evolve through the exploration of new ICPs with tailored redox characteristics and improved stability.

One such polymer is polyindole (PIn), whose backbone combines structural features of poly­(para-phenylene) and polypyrrole.^31,32^ PIn offers a wide accessible redox-potential window, good electrical conductivity, and high thermal and oxidative stability, which make it attractive for sensing applications. Unlike PANI, which typically requires acidic conditions for optimal conductivity, PIn has been reported to retain electrochemical activity under near-neutral conditions, which is essential for biological sensing such as uric acid detection in blood serum. Nevertheless, compared with the more established ICPs PANI, PPy, and PPP, only a few studies have examined PIn as an active sensing material, and its potential in electrochemical sensor platforms remains insufficiently explored.

In this study, we demonstrate a PIn-modified glassy carbon (GC) electrode for the electrochemical quantification of UA. The central objective is to assess whether a thin PIn film enables sufficiently sensitive and selective UA detection in the presence of physiologically relevant interferents such as ascorbic acid, dopamine, and glucose. We hypothesize that the redox-active indole units provide accessible sites that facilitate electron transfer during UA oxidation. To this end, we evaluate the electrochemical response of the PIn/GC electrode toward UA, examine its selectivity against common interferents, and determine the key analytical performance parameters. Overall, this work clarifies the suitability of PIn as a sensing layer and contributes to the limited literature on PIn-based electrochemical sensors.

## 2 Experimental Section

### 2.1 Chemicals and Materials

Potassium hydrogen phosphate (K_2_HPO_4_, 98%), potassium dihydrogen phosphate (KH_2_PO_4_, >99%), indole (C_8_H_7_N, >99%), dodecylbenzenesulfonic acid (DBSA, 95%), ammonium persulfate ((NH_4_)_2_S_2_O_8_, >98%), acetone (C_3_H_6_O, >99%), chloroform (CHCl_3_, >99%) and toluene (C_6_H_5_CH_3_) were purchased from Sigma-Aldrich. 2-Propanol (C_3_H_7_OH) and dimethyl sulfoxide were purchased from Daejung. Sulfuric acid (H_2_SO_4_, 98%), and uric acid (C_5_H_4_N_4_O_3_, 99%) were purchased from Alfa Aesar. All chemicals were used without further purification. Deionized water was used for the electrolyte preparation (Merck Milli-Q, >15 MΩ). Blood serum samples were obtained from Khyber Teaching Hospital (Peshawar City, Pakistan)

### 2.2 Synthesis of Polyindole

Polyindole was synthesized by chemical oxidative polymerization (Scheme 1).^33^ In a 250 mL round-bottom flask, sulfuric acid (40 mL, 0.1 mol L^–1^), DBSA (2 mL, 6.49 mmol, 2.4 equiv), and ammonium persulfate (1.67 g, 7.30 mmol, 2.7 equiv) were added. Polymerization was initiated by addition of indole (0.317 g, 2.70 mmol, 1.0 equiv) and the mixture was stirred vigorously at room temperature for 24 h. Afterward, a 1:1 (v/v) acetone/water mixture was added to precipitate the polymer. The precipitate was filtered and washed with acetone until the filtrate became colorless, then dried in an oven at 60 °C to constant weight, providing polyindole as dark green powder in quantitative yield.

**Scheme 1:**
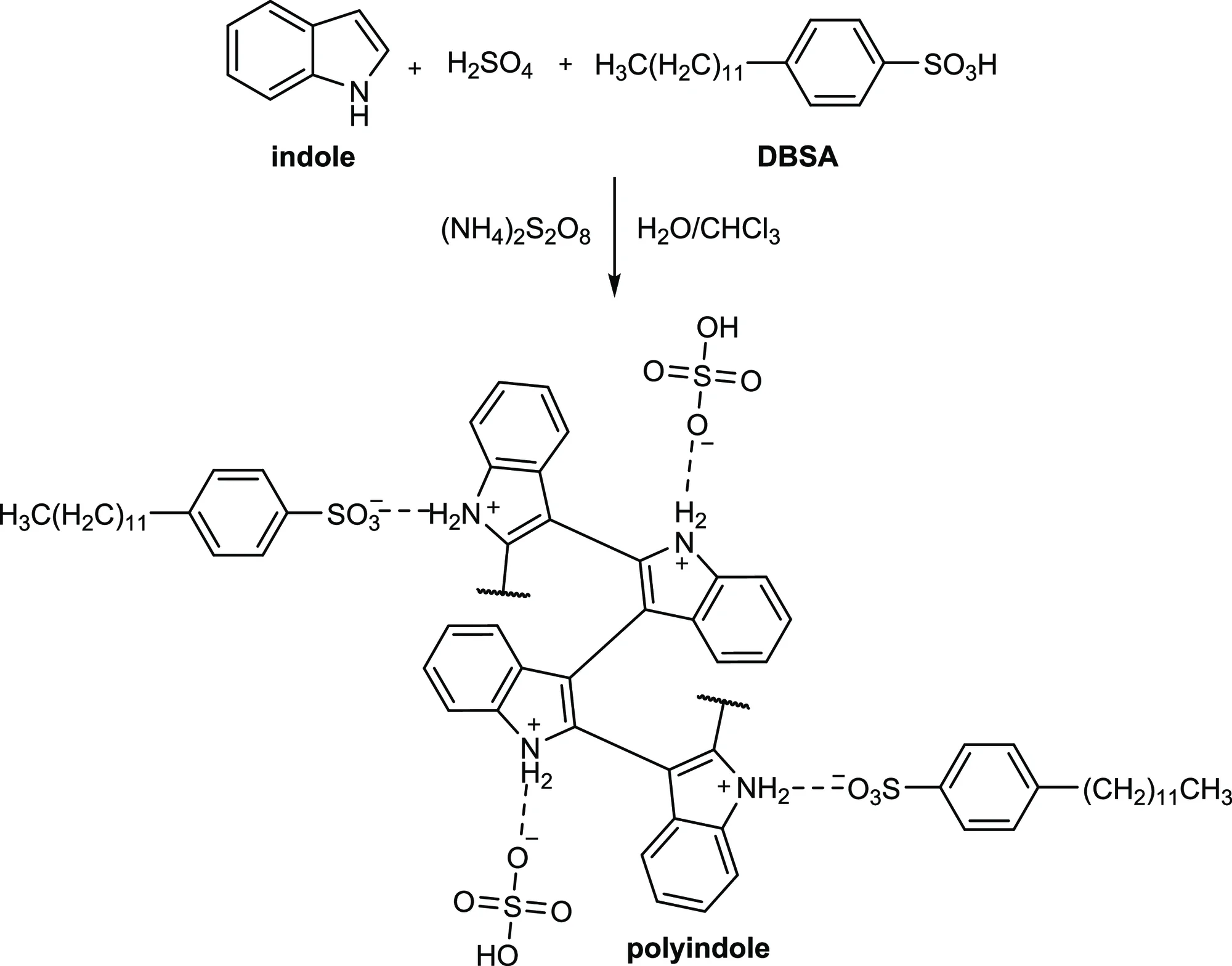
Synthesis and Proposed Polymerization Mechanism of PIn^a^

a The figure was adapted from refs 33, 34 . Copyright 2021 and 2023, MDPI (CC BY 4.0).

### 2.3 Material Characterization

Polyindole morphology and crystalline structure were examined using a scanning electron microscope (SEM, Supra 55VP, ZEISS FEGSEM) with a field-emission electron gun (FEG). The sizes of the recorded structures were evaluated using the program ImageJ 1.46. The elemental composition and homogeneity of polyindole were analyzed with an integrated Oxford Instruments (Oxford Instruments, Abingdon, UK). For investigation of the structural characteristics and functional groups, a Fourier-transform infrared spectrometer (FTIR, Cary 630, Agilent Technologies) was used. Spectra were recorded in attenuated total reflection (ATR) mode between wavenumbers of 400–3500 cm^–1^ with a resolution of 2 cm^–1^. X-ray diffraction (XRD) was performed using the Bruker D8 ADVANCE diffractometer with Cu Kα radiation (λ = 1.54 Å) generated at 40 kV and 40 mA. The intensity of the scattered X-rays was measured in a 2θ-range of 10–70° with step sizes of 0.01° and 2 steps/s. The average crystallite size *D* (nm) was calculated using Debye–Scherrer’s eq 1:

1 $$D=\frac{K\times \lambda }{\beta \times \cos\theta }$$

Here, *K* is the Scherrer constant, having the value of 0.9, β is the half peak width (rad), λ is the wavelength of Cu Kα radiation (1.54 Å), and θ is the diffraction angle. Optical absorption properties were investigated using a UV–vis spectrophotometer (PerkinElmer) in the range of 200–950 nm. Thermogravimetric analysis (TGA) was performed on a STA8000 (PerkinElmer).

### 2.4 Surface Modification of the Glassy Carbon Electrode

The surface of the glassy carbon electrode was mechanically polished on a diamond polishing cloth with Al_2_O_3_ suspension. The electrode was then sonicated in deionized water for 5 min and thoroughly rinsed with deionized water to remove residual alumina. Subsequently, the GC electrode was electrochemically activated by potential cycling between −0.3 and 0.3 V at a scan rate of 100 mV s^–1^ in 0.5 M H_2_SO_4_ until stable cyclic voltammograms were obtained.

For surface modification, 2 mg of PIn were dispersed in 1.0 mL of toluene/2-propanol (2:1 v/v) by ultrasonication for 15 min to obtain a homogeneous suspension. An aliquot of 8.0 μL of this suspension was drop-cast onto the cleaned and activated GC electrode and dried at 25 °C in air.

### 2.5 Determination of the Electrochemically Active Surface Area

The electrochemically active surface area (ECSA) of the uncoated glassy carbon (GC) and polyindole (PIn)-modified GC electrodes was determined from the electrochemical double-layer capacitance (C_DL_). Measurements were carried out in a three-electrode cell with the uncoated or PIn-modified GC electrode as the working electrode, an Ag/AgCl (KCl_sat_ in H_2_O) reference electrode, and a gold sheet (1 cm^2^) as the counter electrode, in 0.5 M H_2_SO_4_ at 25 °C. To determine the potential range, open circuit potential measurements (OCP) of each sample were conducted, and the double-layer capacitance was measured by CV in a potential range of ±5 mV of the OCP at varying scan rates (10, 30, 50, 70, 90, 100 mV s^–1^) going from high scan rates to low scan rates. From the CVs, the current was extracted in the midpoint of the potential window, and the double-layer capacitance C_DL_ was obtained from the slope of the current as a function of scan rates.^35^ The ECSA was calculated by dividing the C_DL_ of the coated electrode by the specific capacitance C_S_ of the uncoated GC electrode as a reference, assuming that the number of active sites is linearly proportional to the C_DL_. Finally, the roughness factor (RF) was obtained by dividing the ECSA by the geometric surface area of the electrode.

### 2.6 Electrochemical Sensing of Uric Acid

Phosphate buffer solutions (PBS, 0.1 M) were prepared from K_2_HPO_4_ and KH_2_PO_4_ by adjusting their ratio according to the Henderson–Hasselbalch equation; unless stated otherwise, measurements were performed at pH 7.0. A 0.01 M stock solution of UA was prepared by dissolving 84.0 mg of UA in 10 mL of 0.02 M NaOH. The basic conditions facilitated complete dissolution of UA; the solution was ultrasonicated and then diluted with 0.1 M PBS to obtain the desired concentrations. This standard solution was used both to construct calibration plots in PBS and to spike human serum samples in the standard addition experiments. Human serum samples were obtained with institutional approval and informed consent in accordance with applicable guidelines.

Electrochemical measurements for UA detection were performed in a three-electrode configuration using the bare or PIn-modified GC electrode as the working electrode, a gold coil as the counter electrode, and an Ag/AgCl (KCl_sat_ in H_2_O) reference electrode. Currents were normalized to the ECSA of the electrode to obtain current densities $j\;({\mathrm{mA cm}}_{\mathrm{ECSA}}^{-2})$ .

Cyclic voltammetry measurements were conducted at a scan rate of 50 mV s^–1^ (unless otherwise stated) over a potential window Δ*E* from −0.2 to +0.8 V vs Ag/AgCl at 25 °C.

The selectivity of the sensor toward UA was evaluated by chronoamperometry. For calibration chronoamperometric current densities were recorded at an applied potential of 0.5 V vs Ag/AgCl in 0.1 M PBS (pH 7.0) for a series of UA concentrations. After each addition of UA, the current was allowed to reach a quasi-steady-state value, which was used for further analysis. Calibration curves were calculated by plotting the steady-state current density *j* as a function of UA concentration *c* (mM) and fitting the data by linear regression.

The linear range was defined as the concentration interval over which the regression coefficient R^2^ remained high (>0.98) and no systematic deviation from linearity was observed. The sensitivity *S* of the sensor was obtained from the slope of the linear calibration plot of current density vs UA concentration (d*j*/d*c*) and is reported in units of ${\mathrm{\mu A cm}}_{\mathrm{ECSA}}^{-2}\;{\mathrm{mM}}^{-1}$ .

The limit of detection (LOD) for UA was calculated from the slope of the chronoamperometric calibration curve and the standard deviation of the intercept of the current density (σ). According to the 3σ criterion, the LOD was obtained as (eq 2):^36^

2 $$\mathrm{LOD}=\frac{3\sigma }{m}$$

Where σ is the relative standard deviation of the current density measured in the absence of UA (blank) and *m* is the slope of the calibration plot.

The relative standard deviation (RSD) was calculated as (eq 3):

3 $$\mathrm{RSD}\left(\%\right)=\frac{s}{\mathrm{x̅}}\times 100$$

where *s* is the standard deviation and x̅ is the mean value of the corresponding current density.

Repeatability and reproducibility were assessed from peak current densities *j*_p_ of CVs measured under identical conditions from five consecutive measurements with the same PIn-modified GC electrode, whereas between-electrode reproducibility was determined from measurements with five independently prepared PIn-modified GC electrodes.

For the stability tests, the current density response to UA was monitored for different durations at 0.5 V vs Ag/AgCl. The relative change in sensor response was expressed as the ratio of the current density at time *t*, *j*(*t*), to the initial current density, *j*_0_ (eq 4):

4 $$\mathrm{Relative}\;\mathrm{response}\;\left(\%\right)=\frac{j\left(t\right)}{j_{0}}\times 100$$

## 3 Results and Discussion

### 3.1 Analysis of the Physicochemical Properties of Polyindole

The synthesized PIn was characterized by FTIR, XRD, SEM/EDX, UV–vis spectroscopy, and thermogravimetric analysis to characterize its physicochemical properties. The results are consistent with the expected structure, doping state, morphology, and thermal stability of PIn.

The FTIR spectrum of PIn (Figure 1a) shows the characteristic vibrational features of the indole-based conjugated backbone together with bands arising from the incorporated dopant species.^15,30,33,34,36,37^ The main band positions and assignments are summarized in Table 1.

**Figure 1:**
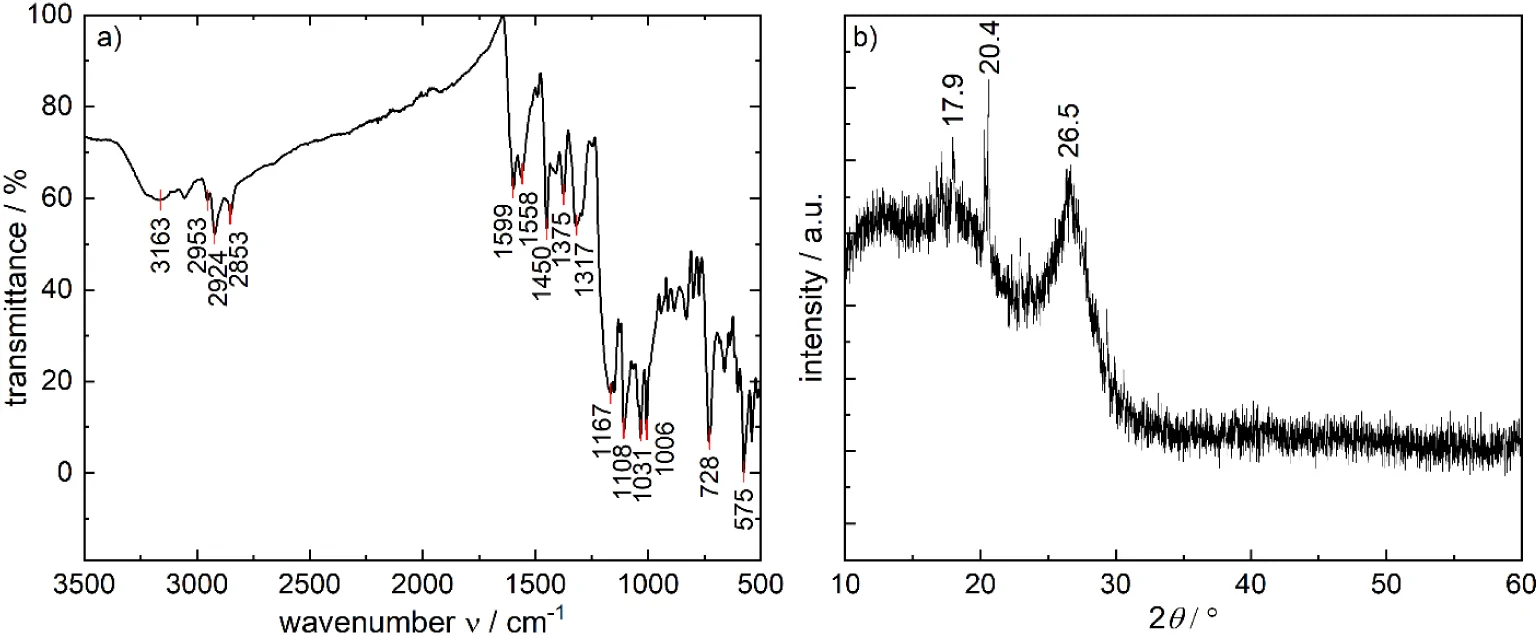
Analysis of PIn by (a) FTIR spectroscopy and (b) X-ray diffraction.

**Table 1:** Attribution of the Key IR Bands of PIn

Wavenumber (s)	Vibration
3163 cm^–1^	ν (N–H)
2953–2853 cm^–1^	ν (C–H_arom_), ν (C_aliphatic_–H (DBSA))
1599 cm^–1^	ν (C=C_arom_, C–C_arom_)
1558 cm^–1^	δ (N–H)
1450 cm^–1^	ν (C–N)
1317 cm^–1^	ν (C–C_arom_)
1167 cm^–1^	$\nu \;({\mathrm{HSO}}_{3}^{-}/{\mathrm{HSO}}_{4}^{-}\;(H_{2}{\mathrm{SO}}_{4}/\mathrm{DBSA}))$
1108 cm^–1^	ν (C–N)
1031 cm^–1^	ν (C–H (DBSA))
1006 cm^–1^	ν (S=O)
728 cm^–1^	γ (C–H_out‑of‑plane_)
575 cm^–1^	$\delta ({\mathrm{HSO}}_{3}^{-}\;(\mathrm{DBSA}))$

Bands associated with the PIn backbone include the N–H stretching vibration at 3163 cm^–1^, aliphatic and aromatic C–H stretching modes in the region 2953–2853 cm^–1^ (together with DBSA alkyl chain), and the C–C/C=C vibrations of the aromatic rings at 1599 cm^–1^. The N–H deformation band at 1558 cm^–1^, the C–N stretching at 1450 cm^–1^, and the band at 1317 cm^–1^ attributed to C–C vibrations in the aromatic ring further support the formation of an indole-based conjugated polymer. Additional contributions from C–N stretching around 1108 cm^–1^ and C–H out-of-plane deformation at 728 cm^–1^ are also consistent with the expected structure of PIn.

The incorporation of the dopant acids is evidenced by several bands assignable to hydrogen sulfate and sulfonate species originating from H_2_SO_4_ and DBSA. A band at 1167 cm^–1^ is attributed to hydrogen sulfate-related vibrations (HSO_4_^–^ /HSO_3_^–^), while the band at 1006 cm^–1^ corresponds to S=O stretching. The signal at 575 cm^–1^ is assigned to HSO_3_^–^ vibrations of DBSA, and the band at 1031 cm^–1^ arises from C–H modes of the DBSA moiety. Together, these features confirm the presence of a doped PIn structure in which the indole-based conjugated backbone is protonated and charge-balanced by sulfonate and hydrogen sulfate counterions.

The X-ray diffractogram of PIn (Figure 1b) displays three broad diffraction peaks at 2θ ≈ 17.5°, 20.4°, and 26.8°, indicating a mixed amorphous/partially crystalline structure.^33,34^ Such broad reflections are typical for doped intrinsically conducting polymers, which show short-range ordering along the polymer chains superimposed on an overall amorphous matrix. The average crystallite size, calculated using the Debye–Scherrer equation, is approximately 14 nm, consistent with a nanocrystalline, short-range-ordered material.

The morphology of PIn was examined by SEM (Figure 2a). The images reveal a granular, approximately spherical morphology with relatively uniform particles and an average size in the range of 100 nm. At higher magnification (Figure 2b), the surface appears rough and composed of smaller subunits; the ∼100 nm particles thus represent aggregates of the much smaller (∼14 nm) coherently diffracting domains derived from XRD. This nanoscale, rough morphology is expected to provide an increased electrochemically active surface area, which may benefit electrochemical charge transfer and, hence, sensing performance.

**Figure 2:**
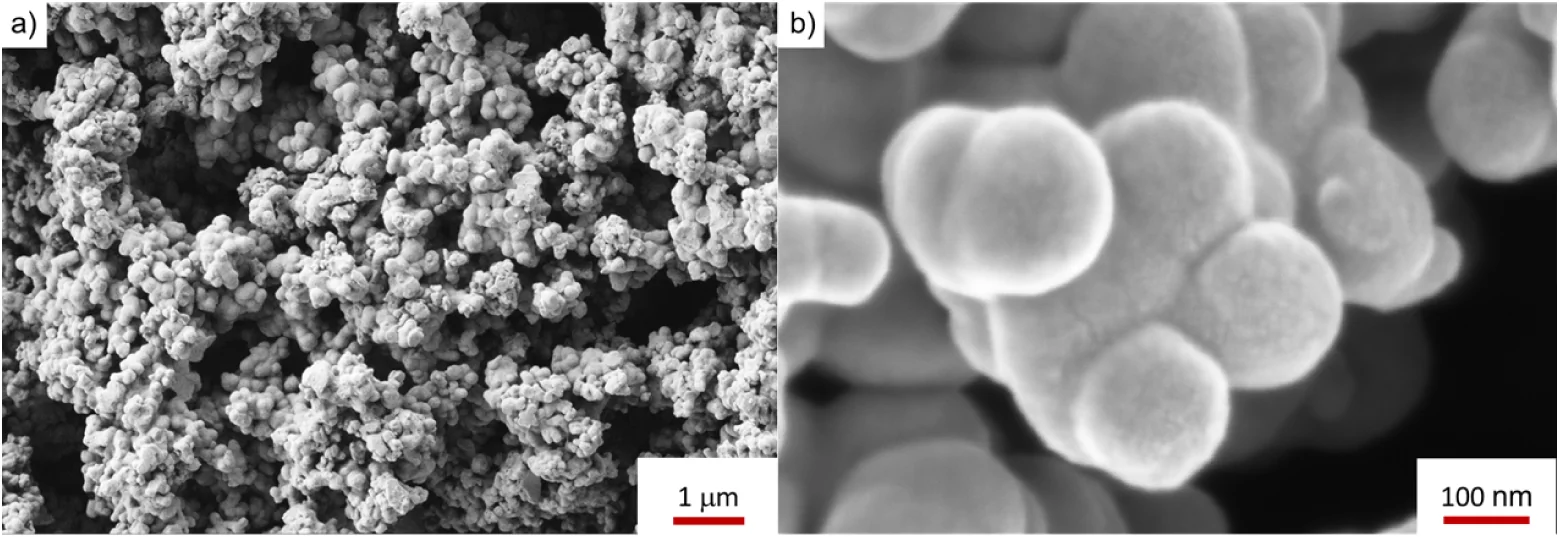
SEM images of polyindole at different magnifications.

The elemental composition and distribution of PIn were further analyzed by EDX (Figure S1). Besides carbon and nitrogen, which are the main constituents of the polyindole backbone, oxygen and sulfur are also detected. The presence of sulfur and oxygen is consistent with the incorporation of sulfonate and hydrogen sulfate dopant species originating from DBSA and H_2_SO_4_. The quantitative elemental composition (Table S1) shows a high carbon content together with detectable amounts of nitrogen, oxygen, and sulfur, in agreement with the proposed doped PIn structure.^33,34^

The UV–Vis absorption spectrum of PIn shows several characteristic bands of the conjugated polymer (Figure S2).^33,34,37^ An absorption band at around ∼274 nm is attributed to π–π* transitions in localized aromatic segments, and a more intense band at approximately 307 nm is associated with π–π* transitions of the conjugated benzene rings in the PIn units. Additional, broader bands in the range of about 350–400 nm (with peaks around 350, 376, and 398 nm) are ascribed to polaronic excitations and π–π*/n−π* electronic transitions in the doped polymer backbone, reflecting the presence of charge carriers generated upon protonic doping. These features are consistent with the formation of a conjugated, doped PIn network.

The thermal stability of PIn was evaluated by TGA (Figure S3). A first weight loss of about 6% above ∼120 °C is attributed to the evaporation of physically adsorbed water, residual solvent, and low-molecular-weight species entrapped in the hydrophilic polymer matrix.^37,38^ A second mass loss of about 80% between 250 and 630 °C is attributed to dedoping and to the thermal degradation of the dopant and the PIn backbone.^39^ No significant further mass loss occurs between 630 and 900 °C; >900 °C, all remaining residue decomposes.

Taken together, the spectroscopic, structural, and thermal data indicate the formation of a doped, nanostructured, and thermally stable PIn with a surface area and accessible charge carriers. These characteristics provide a suitable basis for its use as an electroactive coating on glassy carbon electrodes for the electrochemical quantification of uric acid, as investigated in the following sections.

### 3.2 Electrochemical Characterization of PIn/GC Electrodes

To evaluate the suitability of PIn as an electroactive coating for uric acid sensing, the electrochemical behavior of a glassy carbon (GC) and PIn-modified GC electrode was investigated with respect to electroactive surface area and redox behavior. The electrochemical double-layer capacitance (C_DL_) measured by cyclic voltammetry in the non-Faradaic potential area was used to determine the ECSA (Figure S4). For the PIn-modified GC electrode, the ECSA was 43.64 cm^2^ (R_F_ ≈ 43.6) compared with a 1 cm^2^ electrode area of bare GC. This substantial increase provides additional electroactive sites for charge transfer, which is favorable for the electrochemical detection of uric acid.

For comparison, cyclic voltammograms of the bare GC electrode and the PIn-modified GC electrode were recorded in 1 M H_2_SO_4_ at 25 °C. The bare GC electrode shows a nearly featureless, rectangular response with a low background current, indicating the absence of pronounced faradaic processes and reflecting the limited electroactive surface area and comparatively slow electron transfer of the unmodified electrode (Figure 3a). In contrast, the PIn-modified GC electrode exhibits a well-defined redox couple with anodic and cathodic peaks at approximately 0.62 and 0.14 V, respectively. This couple reflects the quasi-reversible transition between different oxidation states of the doped PIn film (Figure 3b).^33,34,37,38^

**Figure 3:**
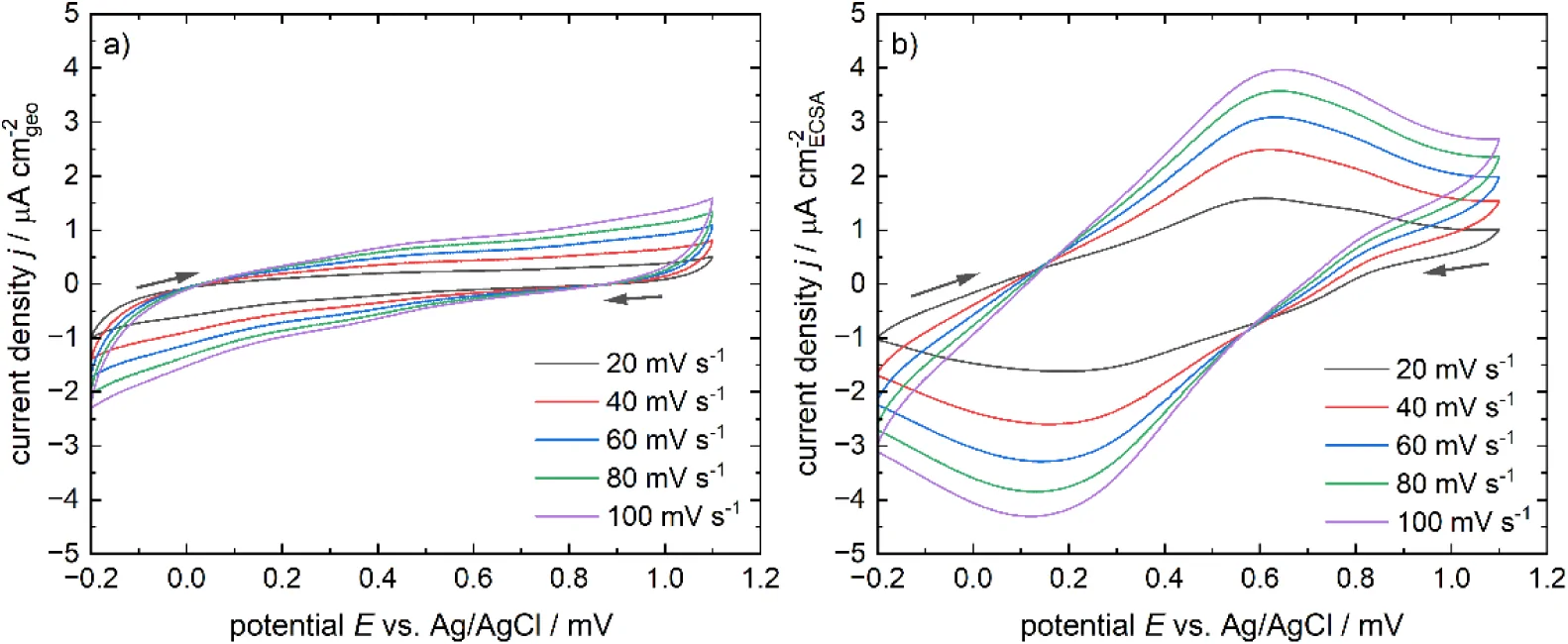
Cyclic voltammograms of (a) the bare GC electrode and (b) the PIn-modified GC electrode recorded in 1 M H_2_SO_4_ at 25 °C at different scan rates.

As discussed above, PIn is obtained in a doped state containing HSO_4_^–^ anions (from H_2_SO_4_) and bulky DBSA anions. During potential cycling, ions from the electrolyte (H^+^, SO_4_^2–^/HSO_4_^–^) can exchange with these counterions in the polymer matrix. The higher mobility of the sulfate species compared with the sterically hindered DBSA anions facilitate reversible doping/dedoping, whereas the more strongly bound DBSA anions contribute to the structural stability of the PIn film.^33,34^

The appearance of sharp and reproducible redox peaks together with the markedly increased current response demonstrates that the PIn coating substantially enhances the electrochemical activity of the GC electrode and provides an electroactive interface suitable for sensing applications. The anodic and cathodic peak currents scale linearly with the square root of the scan rate rather than with the scan rate itself, with a log–log slope of 0.57–0.61 (close to the theoretical 0.5). This indicates a predominantly diffusion-controlled redox process (Figure S6).

### 3.3 Electrochemical Sensing of Uric Acid

Conducting polymers have been widely employed as electrode coatings for the electrochemical oxidation and quantification of uric acid.^17,40^ Building on the material and electrochemical characterization of the PIn-film, its sensing behavior toward UA can be rationalized as follows: The highly conjugated π-electron backbone and the protonated nitrogen centers of PIn provide a redox-active, electronically conductive matrix that facilitates interfacial electron transfer. The incorporated dopants contribute complementary roles: the sulfonate groups of DBSA stabilize the charged polymer chains and enhance charge mobility, whereas H_2_SO_4_ protonates the backbone and thereby supports its conductivity. Together with the high ECSA of the film, these features provide an interface favorable for the electro-oxidation of UA.

During sensing, UA diffuses to the electroactive PIn interface, where it is oxidized; π-π stacking, hydrogen bonding, and electrostatic interactions with the conjugated, protonated backbone and dopants may facilitate this interfacial electron transfer. The oxidation proceeds through a 2e^–^/2H^+^ step, ultimately yielding allantoin,^17,40^ and gives rise to an anodic current that increases with UA concentration. In the following, this response is quantified in terms of linear range, sensitivity, limit of detection, selectivity, and stability.

The electro-oxidation of uric acid at the PIn-modified GC electrode was investigated in neutral phosphate buffer to assess its suitability as a sensing interface under physiologically relevant conditions.

First, the cyclic voltammetric behavior of the PIn-modified GC electrode was studied in 0.1 M phosphate buffer solution (pH 7.0) in the absence of uric acid (Figure 4a). The voltammograms display a quasi-reversible redox couple attributable to the PIn film, with current densities that are comparable to those observed in 1 M H_2_SO_4_. This demonstrates that the doped PIn layer remains electrochemically active and conductive at neutral pH. The degree of quasi-reversibility becomes more pronounced at higher scan rates, as reflected by the increasing peak separation between anodic and cathodic sweeps.

**Figure 4:**
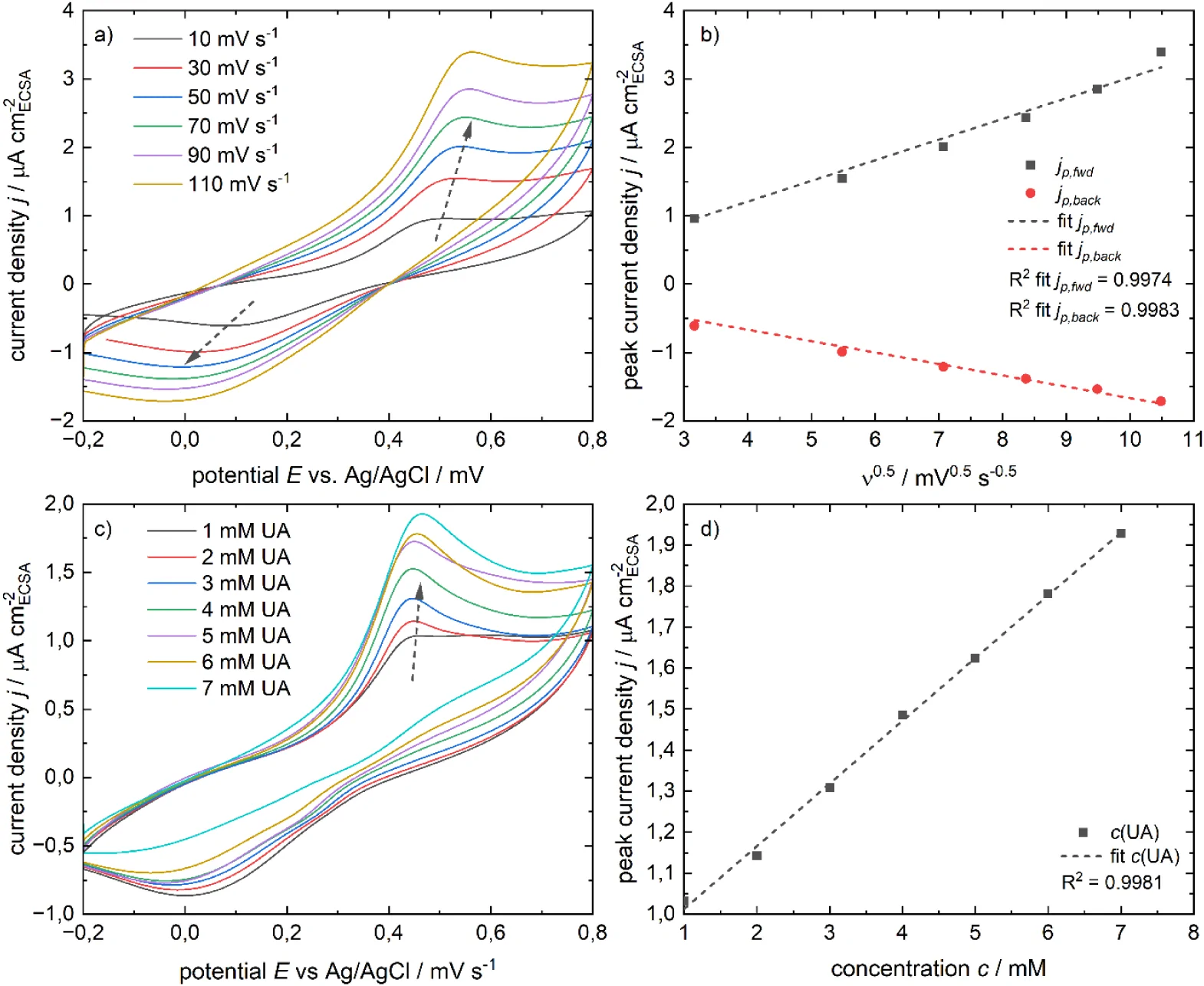
(a) CVs of PIn-modified GC electrode in 0.1 M PBS buffer (pH = 7) at different scan rates; (b) plot of anodic and cathodic peak current densities *j*_p_ vs $\sqrt{\nu }$ ; (c) CV of PIn-modified GC electrode at 50 mV s^–1^ at different UA concentration; and (d) plot of the anodic peak current density *j*_p_ vs UA concentration.

The influence of scan rate on the redox behavior of PIn-modified GC electrode in PBS was analyzed by plotting the anodic and cathodic peak current densities as a function of the square root of the scan rate (Figure 4b). Both peak currents depend linearly on the square root of the scan rate, indicating that the current is diffusion-controlled, in agreement with the behavior in 1 M H_2_SO_4_. The increasing peak separation with scan rate, together with the difference between the anodic and cathodic slopes, further indicates quasi-reversible electron-transfer kinetics of the PIn redox couple, the reduction being somewhat more kinetically hindered than the oxidation.

Upon addition of uric acid to the PBS electrolyte, the quasi-reversible response of the PIn-modified GC electrode changes markedly (Figure 4c). The anodic peak current increases significantly with uric acid concentration, whereas the corresponding cathodic peak shows no proportional change. This behavior is consistent with the irreversible electro-oxidation of uric acid at the PIn-modified GC electrode, superimposed on the quasi-reversible redox transitions of the PIn film. The selective increase of the anodic current in the forward scan reflects the net consumption of uric acid and the absence of a fully reversible back reaction under the applied conditions. The slight change of the cathodic peak with uric acid concentration indicates a minor influence of the oxidation process on the PIn redox chemistry. This can be attributed to the partially reducible uric acid oxidation product and to a transient perturbation of the protonation-dependent doping/dedoping of the pH-sensitive PIn film by the protons released during the 2e^–^/2H^+^ oxidation. As this cathodic contribution is small and the analytical signal is based on the anodic peak current, it does not affect the quantification of uric acid. The sensor response was quantified by plotting the anodic peak current density against uric acid concentration (Figure 4d).

A linear correlation is obtained over the investigated concentration range, showing that the PIn-modified GC electrode responds to uric acid with a proportional current signal and provides a responsive electroactive interface for its quantitative electrochemical sensing.

To evaluate the practical sensing performance, the PIn-modified glassy carbon electrode was operated under potentiostatic conditions in 0.1 M PBS (pH 7.0). Chronoamperometric measurements were performed at different constant potentials *E*, and 0.5 V vs Ag/AgCl was identified as a suitable operating potential that provides a sufficiently high signal for uric acid oxidation while keeping the background current low (Figure S6). At this potential, successive additions of uric acid led to stepwise increases in the chronoamperometric current until quasi-steady-state values were reached, and these steady-state currents were used to quantify the sensor response (Figure 5a). The steady-state current density increased linearly with UA concentration over 0.01–0.10 mM (Figure 5b; R^2^ = 0.998), with a sensitivity of $4.53\;{\mathrm{\mu A cm}}_{ECSA}^{-2}\;{\mathrm{\mu M}}^{-1}$ . Based on the slope of the calibration curve and the standard deviation (3σ criterion, eq 2), the LOD was estimated to be 4.0 μM. A comparison of the analytical performance of the PIn-modified GC electrode with previously reported uric acid sensors is provided in Table S2, showing that the proposed sensor offers a competitive limit of detection and a linear range that, after appropriate sample dilution, covers physiologically relevant uric acid concentrations.

**Figure 5:**
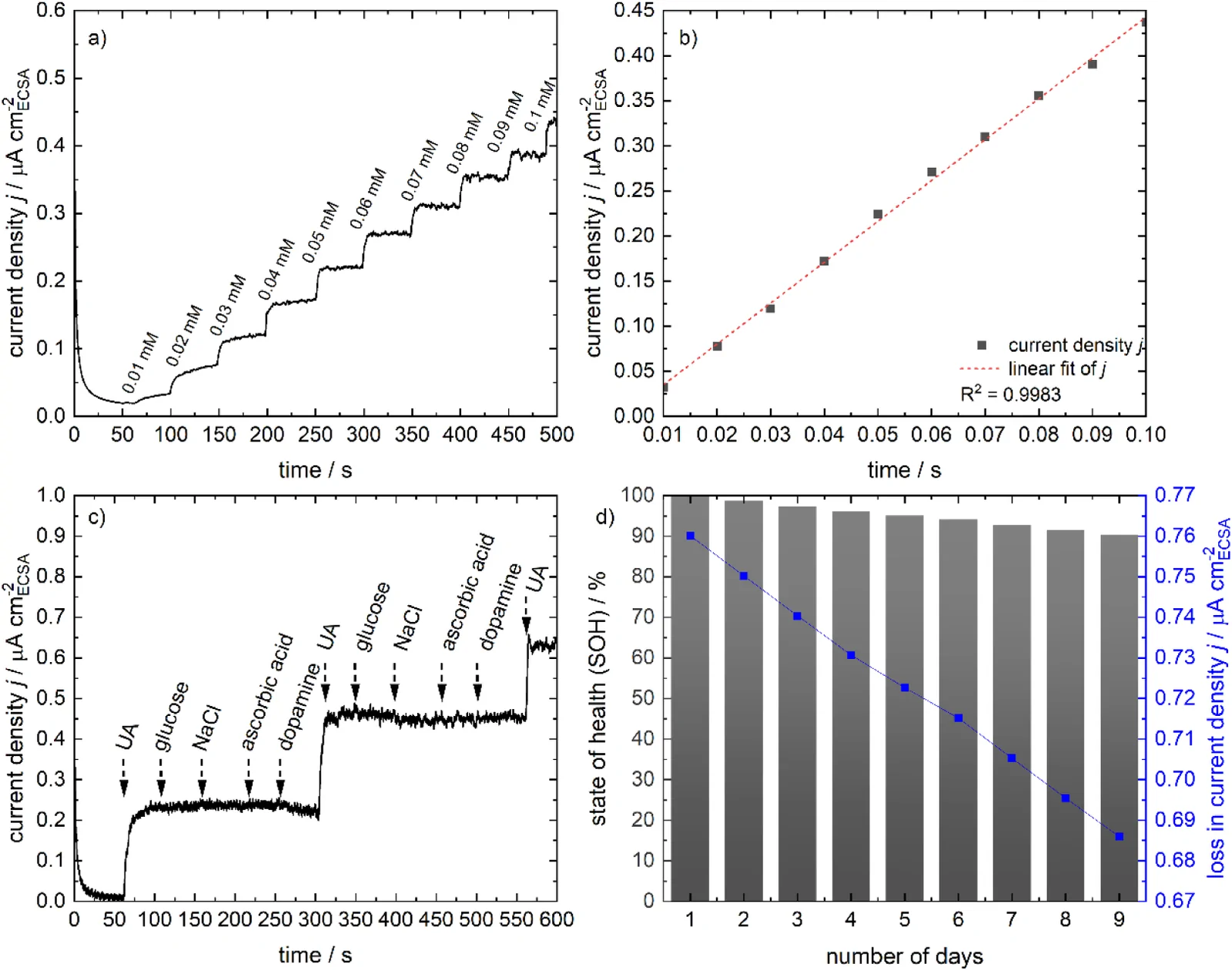
Chronoamperometric sensing of uric acid at the PIn-modified glassy carbon electrode in 0.1 M PBS (pH 7.0) at 0.5 V vs Ag/AgCl: (a) current–time responses recorded for successive additions of uric acid concentrations, (b) corresponding calibration plot of steady-state current density versus uric acid concentration, (c) chronoamperometric response to 0.3 mM uric acid, 5 mM glucose, 0.1 mM ascorbic acid, 0.001 mM dopamine, and NaCl, and (d) long-term stability of the uric acid response over 9 days.

The repeatability of a single PIn-modified GC electrode was evaluated by performing five consecutive amperometric measurements of UA under identical conditions (Table S3 and Figure S7). The steady state currents varied only slightly, corresponding to a relative standard deviation of 2.04%. This indicates that the oxidation products formed under the applied conditions do not significantly foul or degrade the PIn surface and that the electrode can be reused for several measurements without substantial loss of response. The electrode-to-electrode reproducibility was assessed by recording cyclic voltammograms for uric acid oxidation on freshly prepared PIn-modified GC electrodes (Figure S8). The anodic peak currents of five independently prepared electrodes (Table S4 and Figure S9) gave an RSD of 1.01%, indicating that the PIn coating procedure is highly reproducible and yields electrodes with consistent electrochemical performance.

The influence of potentially interfering species on the uric acid response was examined in 0.1 M PBS at 0.5 V vs Ag/AgCl. In plasma, uric acid (≈0.16–0.43 mM) is present at higher concentrations than the other electroactive species dopamine (<0.001 mM) and ascorbic acid (≈0.03–0.10 mM), whereas glucose (3.9–6.1 mM under fasting conditions) and inorganic salts such as NaCl occur at considerably higher levels. These species can nevertheless contribute to the anodic current at the applied potential and may therefore interfere with uric acid detection, particularly at low analyte concentrations. As shown in Figure 5c, the current response of the PIn-modified GC electrode to uric acid at 0.5 V is pronounced, whereas the additional current contributions of glucose, dopamine, ascorbic acid, and NaCl under the same conditions are negligible. This demonstrates that the PIn-modified GC electrode exhibits good selectivity for uric acid over these common interferents in PBS at pH 7.0.

The stability of the PIn-modified GC electrode was assessed both within a single measurement and over several days of storage. In chronoamperometry at 0.5 V in the presence of uric acid, the current density remained essentially constant over 1200 s (Figure S10), indicating a stable response over the time scale of an analytical measurement. Long-term storage stability was evaluated using three independently fabricated electrodes (*n* = 3), stored under dry ambient conditions at room temperature and measured daily over 9 days (Figure 5d, Table 2). Relative to the Day-1 response (defined as 100%), the electrodes showed a gradual, monotonic decrease, retaining 98.7% to 90.3% of their initial response from Day 2 to Day 9—above the acceptance criterion of ≥90% throughout. The low day-to-day RSD across the three electrodes (0.63–1.20%) confirms consistent electrode-to-electrode performance. The gradual decline is attributed to slow dedoping or partial degradation of the PIn film during storage rather than to fouling, since the electrodes were not exposed to analyte between measurements. Overall, the electrode shows acceptable long-term storage stability for practical uric acid sensing.

**Table 2:** Stability of the PIn-Modified GC Electrode in Long-Term Storage *n* = 3 Independently Prepared Electrodes

Day	Mean current density loss/μA cm^–2^	SD	RSD/%	Retention/%
1	0.760	0.21	0.63	100
2	0.750	0.29	0.89	98.7
3	0.740	0.29	0.9	97.4
4	0.730	0.29	0.91	96.13
5	0.723	0.3	0.95	95.08
6	0.715	0.29	0.93	94.08
7	0.705	0.32	1.04	92.78
8	0.695	0.29	0.96	91.49
9	0.686	0.36	1.2	90.25

Selectivity was further evaluated by DPV for uric acid in the presence of common interferents (ascorbic acid, AA; dopamine, DA; glucose, Glc) at physiologically relevant concentrations (Figure 6a). Even in the presence of all interferents, the UA oxidation peak (≈0.43 V vs Ag/AgCl) increased linearly with UA concentration over 0.03–0.24 mM (sensitivity 1.34 μA cm^–2^ mM^–1^, R^2^ = 0.9945; Figure 6b), with no discernible peak shift or distortion and no overlapping peaks. This range reaches physiologically relevant serum levels and indicates that the response arises distinctly from UA.

**Figure 6:**
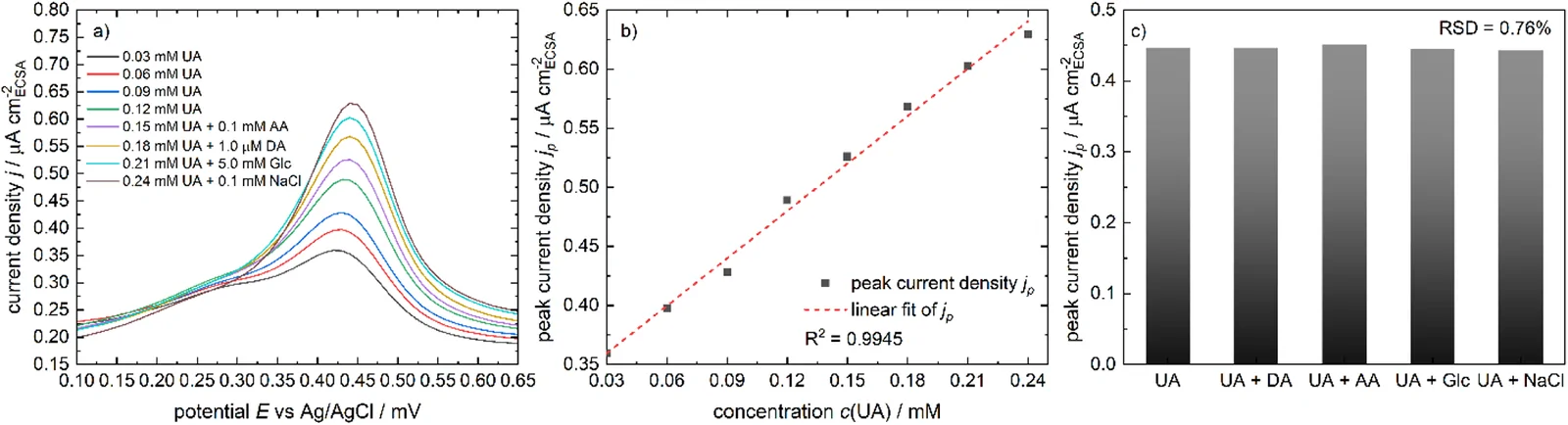
Selectivity of the PIn-modified GC electrode toward UA, assessed by DPV in 0.1 M PBS (pH 7.0). (a) DPV curves for increasing UA concentrations (0.03–0.30 mM) in the presence of the interferents held constant at physiological levels (AA 0.10 mM, DA 0.001 mM, glucose 5 mM, NaCl at physiological level). (b) Corresponding linear fit of the UA peak current density vs UA concentration (linear over 0.03–0.24 mM, R^2^ = 0.9945) with interferents. (c) UA peak current density *j*_p_ in response to 0.30 mM UA in the presence of individual interferents (AA, DA, glucose, and NaCl; *n* = 3) at the tested concentrations.

The selectivity of the electrode was then quantified for individual interferents (AA, DA, Glc, and NaCl). The UA anodic peak currents and the corresponding recoveries (*n* = 3) are summarized in Table 3. In the presence of each interferent, the UA peak current varied only marginally (RSD = 0.76% across all measurements; Figure 6c), with recoveries of 99.1–101.1% and relative errors below 1.1%, confirming no significant interference.

**Table 3:** Recoveries and Interference Effects for the Determination of Uric Acid (0.3 mM) in the Presence of Individual Interferents

Interferents	Interferents level ratio (interferents:uric acid)	*j*_p_ of uric acid (μA cm^–2^)	Recovery (%)
No interferents	-	0.615 ± 0.001	100
UA + DA	1:0.003	0.614 ± 0.001	99.81 ± 0.16
UA + AA	1:0.33	0.621 ± 0.001	101.06 ± 0.21
UA + Glucose	1:16.7	0.611 ± 0.003	99.45 ± 0.45
UA + NaCl	1:∼470	0.609 ± 0.002	99.08 ± 0.31

The capability against interference species was additionally assessed for a multicomponent mixture by comparing the DPV response of 0.30 mM UA alone with that of a mixture containing 0.30 mM UA and all interferents at physiological concentrations (0.10 mM AA, 0.001 mM DA, 5 mM glucose, and NaCl at physiological level) in 0.1 M PBS (pH 7.0). The UA oxidation peak (≈0.44 V) remained essentially unchanged, with a total interference of only 1.3% (Figures S11a and b), indicating that the simultaneous presence of these physiologically relevant species has a negligible effect on UA detection. Overall, these results confirm the high selectivity of the PIn-modified GC electrode and its suitability for uric acid determination in complex matrices.

### 3.4 Determination of Uric Acid in Blood Serum

To assess the practical applicability of the proposed sensor, human blood samples were collected from several patients (with institutional approval and informed consent; Figures S12). The samples were centrifuged to separate the serum and diluted 10-fold with PBS. Endogenous uric acid was determined by the chronoamperometric standard-addition method;^41–43^ the analytical data are summarized in Table 4. Taking the 10-fold dilution into account, the endogenous serum uric acid concentrations ranged from 0.23 to 3.26 mM. Recoveries ranged from 99.68% to 99.92% with RSD values below 5%, indicating acceptable accuracy, precision, and repeatability.

**Table 4:** Determination of UA in Human Blood Serum Using the PIn-Modified GC Electrode and the Chronoamperometric Standard Addition Method

Blood samples^a^,^b^	Baseline Current/μA	Endogenous UA/mM	Spiked Range/mM	Found UA Range/mM	Mean recovery^c^/%	RSD/%
1	0.72 ± 0.02	0.044	0.02–0.10	0.064–0.14	99.82 ± 1.03	2.97
2	0.69 ± 0.012	0.142	0.10–0.50	0.24–0.64	99.74 ± 0.68	1.73
3	3.42 ± 0.06	0.326	0.10–1.00	0.42–1.32	99.92 ± 0.95	1.45
4	3.18 ± 0.07	0.154	0.10–0.60	0.25–0.75	99.80 ± 0.71	2.20
5	6.8 ± 0.28	0.023	0.02–0.07	0.0043–0.093	99.82 ± 0.63	4.12
6	7.10 ± 0.18	0.029	0.02–0.10	0.049–0.13	99.80 ± 0.47	1.12
7	7.55 ± 0.12	0.034	0.02–0.10	0.054–0.13	99.68 ± 0.43	4.10

a Human blood serum; analyte: UA; supporting electrolyte: 0.1 M PBS (pH 7.0).

b Electrochemical conditions: chronoamperometry at 0.5 V vs Ag/AgCl using the PIn-modified GC electrode.

c $\mathrm{Recovery}=\left(c_{\mathrm{found}}-c_{\mathrm{endogenous}}/c_{\mathrm{spiked}}\right)\times 100$ .

Overall, the recoveries close to 100% and RSD values below 5% suggest that matrix effects in diluted human serum are minor and that the calibration established in PBS can be transferred to serum samples. Given the chronoamperometric linear range of 0.01–0.10 mM and the 10-fold sample dilution, physiological uric acid levels (≈0.16–0.43 mM in serum, i.e., ≈0.016–0.043 mM in the diluted sample) fall within the calibrated range, so that the sensor covers the clinically relevant concentration range. Although the present study is limited to a small number of spiked serum samples, the results demonstrate the feasibility of using the PIn-modified GC electrode for uric acid determination in real biological matrices.

## 4 Conclusions

In this study, a PIn-modified GC electrode was developed for the electrochemical quantification of uric acid. The chemically synthesized PIn, doped with dodecylbenzenesulfonate and sulfate species, was shown by FTIR, XRD, SEM/EDX, UV–vis spectroscopy, and thermogravimetric analysis to form a conjugated, nanostructured, and thermally stable polymer film. Its rough, granular morphology and mobile dopant anions yield an electroactive coating that increases the electrochemically active surface area by 43-times and enhances its charge-transfer behavior.

Electrochemical characterization in acidic and neutral electrolytes revealed a well-defined, quasi-reversible and diffusion-controlled redox couple of the PIn film, together with a markedly higher current-density response than the bare GC electrode. Notably, the doped film remained electrochemically active at neutral pH, providing a stable interface for uric acid oxidation in phosphate buffer (pH 7.0). Chronoamperometry gave a linear response over 0.01–0.10 mM with a sensitivity of 4.53 μA cm^–2^ mM^–1^ and a limit of detection of 4.0 μM, while differential pulse voltammetry extended the accessible linear range to 0.03–0.24 mM. The sensor showed good repeatability and reproducibility, with relative standard deviations of 2.0% for repeated measurements with the same electrode and 1.0% for independently prepared electrodes.

The PIn-modified electrode also exhibited high selectivity toward uric acid in the presence of common interferents such as glucose, ascorbic acid, dopamine, and NaCl. Individual interferents caused recoveries of 99.1–101.1% (RSD 0.76%), and a multicomponent mixture at physiological concentrations altered the uric acid signal by only 1.3%. Short-term chronoamperometric measurements gave stable signals over typical measurement times, and the electrode retained more than 90% of its response over 9 days of storage.

In spiked human serum, the sensor achieved recoveries of 99.7–99.9% with relative standard deviations below 5%, indicating minor matrix effects in diluted serum and confirming that the calibration obtained in phosphate buffer can be transferred to real blood serum samples. Overall, these results establish polyindole as a promising yet still underexplored intrinsically conducting polymer for nonenzymatic electrochemical sensing. Its stable redox activity at neutral pH and its robustness against common interferents make the PIn-modified GC electrode an attractive platform for further development toward point-of-care diagnostic applications.

## Supplementary Material



## Data Availability

The data presented in the manuscript are openly available in the KITopen repository at DOI: 10.35097/4c872ct4ezbhs4rt

## Notes

Experimental data from SEM-EDX analysis and elemental mapping, UV–vis analysis, and TGA measurements; ECSA, kinetic analysis of PIn-modified electrodes, identification of the operational potential window for uric acid detection, a comparison of the analytical performance of the PIn-modified electrode with previously reported sensor materials, and data on electrode reusability, reproducibility, short-term stability, interference experiments, and blood sample tests (PDF)

## References

[1] Chelmea L., Badea M., Scarneciu I., Moga M. A., Dima L., Restani P., Murdaca C., Ciurescu D., Gaman L. E. (2023). New Trends
in Uric Acid Electroanalysis. Chemosensors.

[2] Mota F. A., Passos M. L., Santos J. L., Saraiva M. L. M. (2024). Comparative analysis
of electrochemical and optical sensors for detection of chronic wounds
biomarkers: A review. Biosens. Bioelectron..

[3] Wang J., Zhang Y., Tang Q., Zhang Y., Yin Y., Chen L. (2024). Application of antioxidant
compounds in bone defect repair. Antioxidants.

[4] Fathallah-Shaykh S. A., Cramer M. T. (2014). Uric acid and the
kidney. Pediatr.
Nephrol..

[5] Nishan U., Ahmed A., Muhammad N., Shah M., Asad M., Khan N., Ullah F., Ullah R., Ali E. A., Nawaz H., Badshah A. (2024). Uric acid quantification
via colorimetric
detection utilizing silver oxide-modified activated carbon nanoparticles
functionalized with ionic liquid. RSC Adv..

[6] Yan J., Lin H., Xu Y., Yu L., Chen S., Zhang Q., Han L., Ou Y., Zhan M., Wang J., Ke P. (2022). Measurement
of serum uric acid by isotope dilution liquid chromatography tandem
mass spectrometry: Modification of a candidate reference measurement
method and its clinical application. Int. J.
Mass Spectrom..

[7] Sun M., Cui C., Chen H., Wang D., Zhang W., Guo W. (2023). Enzymatic
and Non-Enzymatic Uric Acid Electrochemical Biosensors: A Review. ChemPlusChem.

[8] Wang Q., Wen X., Kong J. (2020). Recent progress
on uric acid detection: a review. Crit. Rev.
Anal. Chem..

[9] Buledi J. A., Ameen S., Memon S. A., Fatima A., Solangi A. R., Mallah A., Karimi F., Malakmohammadi S., Agarwal S., Gupta V. K. (2021). An improved non-enzymatic electrochemical
sensor amplified with CuO nanostructures for sensitive determination
of uric acid. Open Chem..

[10] Shahzad N., Ajmal R., Afzal A. (2023). Non-enzymatic
electrochemical sensors
for accurate and accessible uric acid detection. J. Electrochem. Soc..

[11] Fang L., Zhang Y., Liu Y., Shou J., Liu H., Li L. (2024). Flexible electrochemical sensor for simultaneous determination
of
levodopa and uric acid based on carbon nanotube fibers. Microchem. J..

[12] Thirabowonkitphithan P., Maksuk K., Permpoka K., Jiraseree-Amornkun A., Wattanapanitch W., Nilaratanakul V., Tasca F., Laiwattanapaisal W. (2024). A flexible
non-enzymatic hydrogel-assisted electrochemical sensor: Wireless,
sensitive, and selective uric acid detection for home monitoring. Microchem. J..

[13] Saputra H. A. (2023). Electrochemical
sensors: basic principles, engineering, and state of the art. Monatsh. Chem..

[14] Soleh A., Kanatharana P., Thavarungkul P., Limbut W. (2020). Novel electrochemical
sensor using a dual-working electrode system for the simultaneous
determination of glucose, uric acid and dopamine. Microchem. J..

[15] Dube A., Malode S. J., Alodhayb A. N., Mondal K., Shetti N. P. (2025). Conducting
polymer-based electrochemical sensors: Progress, challenges, and future
perspectives. Talanta Open.

[16] Tajik S., Beitollahi H., Nejad F. G., Shoaie I. S., Khalilzadeh M. A., Asl M. S., Van Le Q., Zhang K., Jang H. W., Shokouhimehr M. (2020). Recent developments in conducting
polymers: Applications
for electrochemistry. RSC Adv..

[17] Văduva M., Baibarac M., Cramariuc O. (2023). Functionalization
of graphene derivatives
with conducting polymers and their applications in uric acid detection. Molecules.

[18] Yamaguchi I., Fujii N., Wang A. (2020). π-Conjugated
polymer with Alloxazine-6,
9-diyl unit in the Main chain: Synthesis, chemical properties, and
sensing ability for metal ions and nucleosides. React. Funct. Polym..

[19] Bilal S., Akbar A., Shah A.-U.-H. A. (2019). Highly selective and reproducible
electrochemical sensing of ascorbic acid through a conductive polymer
coated electrode. Polymers.

[20] Dakshayini B. S., Reddy K. R., Mishra A., Shetti N. P., Malode S. J., Basu S., Naveen S., Raghu A. V. (2019). Role of conducting
polymer and metal oxide-based hybrids for applications in ampereometric
sensors and biosensors. Microchem. J..

[21] Terán-Alcocer Á., Bravo-Plascencia F., Cevallos-Morillo C., Palma-Cando A. (2021). Electrochemical sensors based on
conducting polymers
for the aqueous detection of biologically relevant molecules. Nanomaterials.

[22] Wahyuni W. T., Rahman H. A., Afifah S., Anindya W., Hidayat R. A., Khalil M., Fan B. R., Putra B. R. (2024). Comparison of the
analytical performance of two different electrochemical sensors based
on a composite of gold nanorods with carbon nanomaterials and PEDOT:
PSS for the sensitive detection of nitrite in processed meat products. RSC Adv..

[23] Prathap M. U., Srivastava R. (2013). Tailoring properties of polyaniline for simultaneous
determination of a quaternary mixture of ascorbic acid, dopamine,
uric acid, and tryptophan. Sens. Actuators,
B.

[24] Sajid H., Mahmood T., Ayub K. (2018). High sensitivity
of polypyrrole sensor
for uric acid over urea, acetamide and sulfonamide: a density functional
theory study. Synth. Met..

[25] Ponnaiah S. K., Periakaruppan P., Vellaichamy B. (2018). New electrochemical sensor based
on a silver-doped iron oxide nanocomposite coupled with polyaniline
and its sensing application for picomolar-level detection of uric
acid in human blood and urine samples. J. Phys.
Chem. B.

[26] Hsine Z., Blili S., Milka R., Dorizon H., Said A. H., Korri-Youssoufi H. (2020). Sensor based on redox conjugated
poly (para-phenylene)
for the simultaneous detection of dopamine, ascorbic acid, and uric
acid in human serum sample. Anal. Bioanal. Chem..

[27] Putra B. R., Nisa U., Heryanto R., Rohaeti E., Khalil M., Izzataddini A., Wahyuni W. T. (2022). A facile electrochemical sensor based
on a composite of electrochemically reduced graphene oxide and a PEDOT:
PSS modified glassy carbon electrode for uric acid detection. Anal. Sci..

[28] Putra B. R., Nisa U., Heryanto R., Khalil M., Khoerunnisa F., Ridhova A., Thaha Y. N., Marken F., Wahyuni W. T. (2022). Selective
non-enzymatic uric acid sensing in the presence of dopamine: electropolymerized
poly-pyrrole modified with a reduced graphene oxide/PEDOT: PSS composite. Analyst.

[29] Safitri H., Wahyuni W. T., Rohaeti E., Khalil M., Marken F. (2022). Optimization
of uric acid detection with Au nanorod-decorated graphene oxide (GO/AuNR)
using response surface methodology. RSC Adv..

[30] Dzulkurnain N. A., Mokhtar M., Rashid J. I. A., Knight V. F., Wan Yunus W. M. Z., Ong K. K., Mohd Kasim N. A., Mohd Noor S. A. (2021). A review
on impedimetric and voltammetric analysis based on polypyrrole conducting
polymers for electrochemical sensing applications. Polymers.

[31] Pandey P. C., Tiwari A. K., Narayan R. J. (2024). Optimization
of solvents, electrolytes,
and mediators for polyindole-based electrochemical sensors. Sens. Diagn..

[32] Thadathil A., Pradeep H., Joshy D., Ismail Y. A., Periyat P. (2022). Polyindole
and polypyrrole as a sustainable platform for environmental remediation
and sensor applications. Mater. Adv..

[33] Begum B., Bilal S., Shah A. U. H. A., Röse P. (2021). Physical,
Chemical, and Electrochemical Properties of Redox-Responsive Polybenzopyrrole
as Electrode Material for Faradaic Energy Storage. Polymers.

[34] Humayun H., Begum B., Bilal S., Shah A. U. H. A., Röse P. (2023). Polyindole embedded nickel/zinc oxide
nanocomposites
for high-performance energy storage applications. Nanomaterials.

[35] Bard, J. ; Faulkner, L. R. Electrochemical Methods: fundamentals and Applications, 2nd ed.; John Wiley & Sons, 2001.

[36] Allegrini F., Olivieri A. C. (2014). IUPAC-Consistent Approach to the
Limit of Detection
in Partial Least-Squares Calibration. Anal.
Chem..

[37] Inamuddin I., Shakeel N., Ahamed M. I., Kanchi S., Kashmery H. A. (2020). Green synthesis
of ZnO nanoparticles decorated on polyindole functionalized-MCNTs
and used as anode material for enzymatic biofuel cell applications. Sci. Rep..

[38] Barde R. V., Nemade K. R., Jagtap K. D., Rathod D. P., Waghuley S. A. (2023). Preparation
of Polyindole-MnO2/Reduced Graphene Oxide-based Ternary Composite
System for Supercapacitive Application. Polym.
Plast. Technol. Eng..

[39] Phasuksom K., Sirivat A. (2016). Synthesis of nano-sized polyindole
via emulsion polymerization
and doping. Synth. Met..

[40] Younis S., Liaqat F., Jabeen A., Ahmed S. (2024). An effective sensing
platform composed of polyindole based ternary nanocomposites for electrochemical
detection of ascorbic acid. Mater. Chem. Phys..

[41] Özcan A., Şahin Y. (2010). Preparation
of selective and sensitive electrochemically
treated pencil graphite electrodes for the determination of uric acid
in urine and blood serum. Biosens. Bioelectron..

[42] Wang Y. K., Chen Y. J., Pan Y., Hai-Yin Y. (2025). Prussian Blue and Polyvinylpyrrolidone-Embedded
Hollow Fiber Gradient Polysulfone Membrane for Uric Acid Biosensor
Application. J. Appl. Polym. Sci..

[43] Abbas Y., Ali S., Basharat M., Zou W., Yang F., Liu W., Zhang S., Wu Z., Akhtar N., Wu D. (2020). Heteroatom-doped
carbon nanoparticle–ionic liquid composites as electrochemical
sensors for uric acid. ACS Appl. Nano Mater..

