# Supplementary material for: Redox-Active Polyindole-Modified Electrode for Electrochemical Uric Acid Determination
Source: ACS Omega. 2026 Sep 2;11(36):54762–72. doi: 10.1021/acsomega.6c08422 (PMC13584534; doi:10.1021/acsomega.6c08422)
Supplement: Supplementary file 1 [file ao6c08422_si_001.pdf]

Supporting Information

Redox-Active Polyindole-Modified Electrode for Electrochemical Uric Acid Determination

Alam Zeb[a], Salma Bilal[a,*], Bushra Begum[a,b], Anwar ul Haq Ali Shah[c], Philipp Röse[d,*]

[a] *National Centre of Excellence in Physical Chemistry, University of Peshawar, 25120 Peshawar, Khyber Pakhtunkhwa, Pakistan*

[b] *Department of Chemistry, Women University, 23200 Mardan, Pakistan*

[c] *Institute of Chemical Sciences University of Peshawar, 25120 Peshawar, Khyber Pakhtunkhwa, Pakistan*

[d] *Institute for Applied Materials – Electrochemical Technologies, Karlsruhe Institute of Technology, Adenauerring 20b, 76131 Karlsruhe, Germany*

*Corresponding authors: salmabilal@uop.edu.pk (S. Bilal), philipp.roese@kit.edu (P. Röse)

Content

1. EDX Spectrum of Polyindole (PIn) 2
2. UV-Vis Analysis of Polyindole 3
3. Thermogravimetric analysis of Polyindole 4
4. Quantification of the electrochemically active surface area (ECSA) 5
6. Identification of the Potential Window for Chronoamperometric Detection of UA on PIn-modified GC electrodes 7
7. Comparison of the analytical performance of the PIn-modified glassy carbon electrode with previously reported uric acid sensors 8
8. Reusibility of PIn-modified GC Electrodes for UA Detection 9
9. Reproducebility of PIn-modified GC Electrodes for UA Detection 10
10. Short-term Stability of PIn-modified GC Electrodes 11
11. References 14

1. EDX Spectrum of Polyindole

Polyindole (PIn) was analyzed by SEM–EDX to confirm the presence and distribution of the elements associated with the polymer backbone and the dopant species. The EDX mapping shows a homogeneous distribution of carbon, nitrogen, oxygen, and sulfur within the probed area (Figure S1), and the quantitative composition is summarized in Table S1.

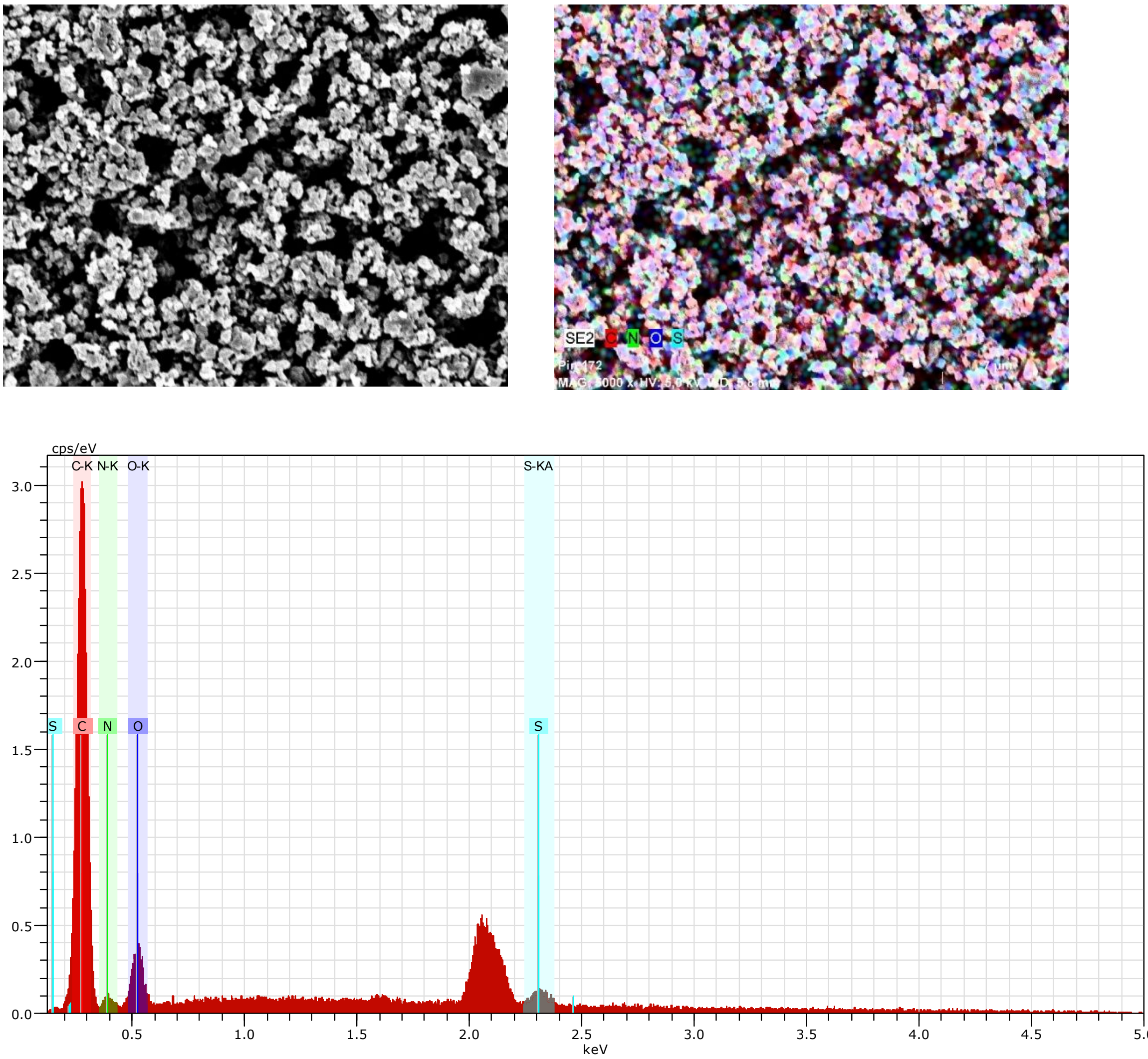

Figure S1. SEM–EDX analysis of PIn: investigated area (top left), elemental maps of C, N, O, and S (top right), and relative atomic composition obtained from the analyzed region (bottom).

Table S1. Elemental composition of PIn.

element[a]	normalized composition / wt.%	atomic composition / at.%	error / %
C	76.30	80.67	23.0
O	12.93	10.26	4.0
N	9.41	8.53	2.9
S	1.35	0.53	0.1

[a] Values obtained from the EDX analysis of the area shown in Figure S1.

2. UV-Vis Analysis of Polyindole

The optical absorption properties of PIn were examined by UV–Vis spectroscopy to support the assignment of the conjugated polymer structure and the presence of doped states. The full absorption spectrum is shown in Figure S2.

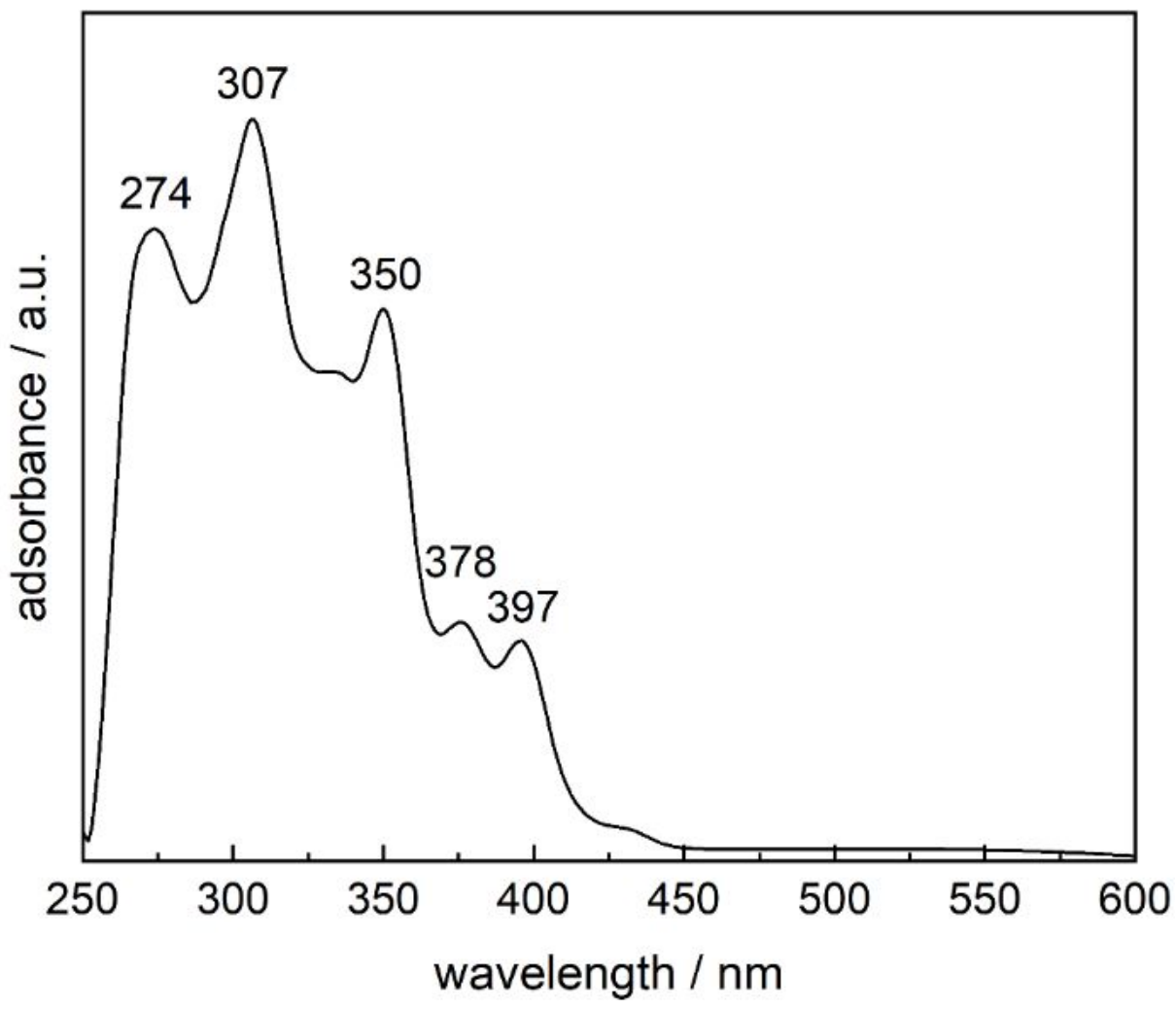

Figure S2. UV–Vis absorption spectrum of PIn recorded in the range 200–900 nm.

3. Thermogravimetric Analysis of Polyindole

The thermal stability and decomposition behavior of PIn were further investigated by thermogravimetric analysis (TGA). The TGA curve shown in Figure S3 complements the discussion in the main text and illustrates the multi-step mass loss profile of the doped polymer.

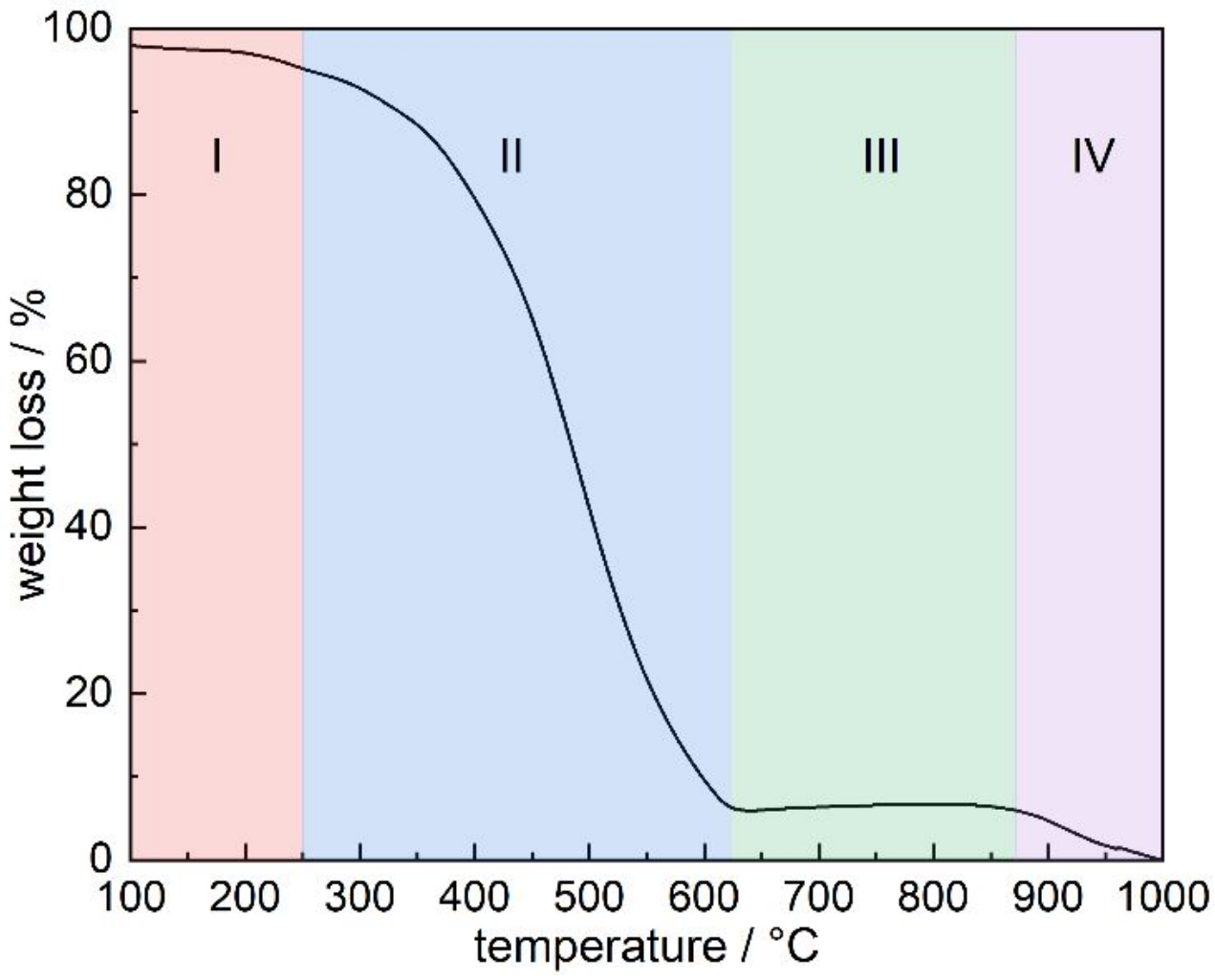

Figure S3. TGA of PIn under N_2 and at heating ramp of 5 K min^{-1}.

4. Quantification of the electrochemically active surface area (ECSA)

The electrochemically active surface area (ECSA) of the bare and PIn-modified glassy carbon (GC) electrodes was determined from electrochemical double-layer capacitance (C_{DL}) measurements.

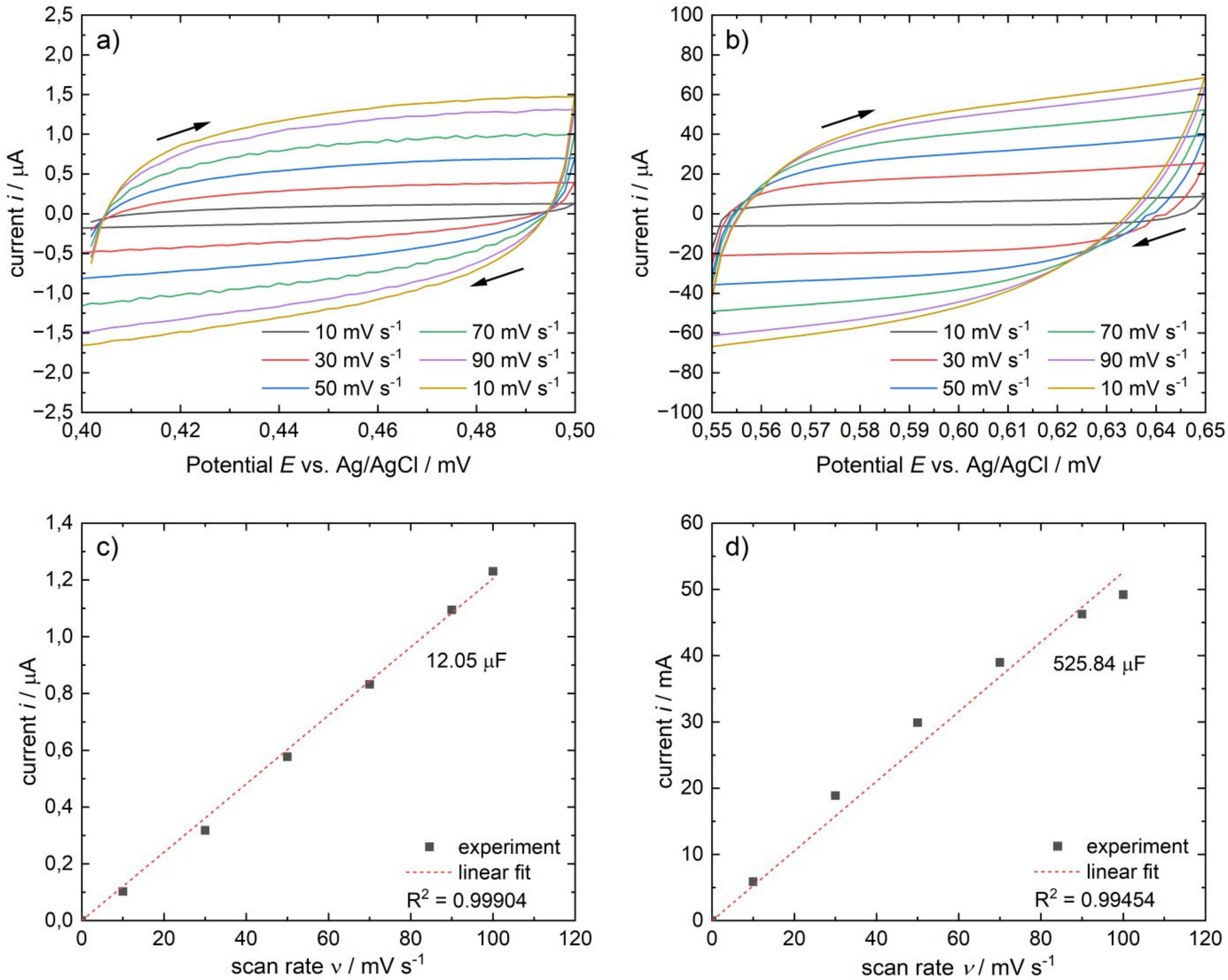

Figure S4. Cyclic voltammograms of the a) GC electrode, and b) PIn-modified GC electrode recorded in 0.5 M H_2SO_4 within the non-Faradaic potential window (0.55-0.65 V) at different scan rates. The corresponding plots of the double-layer current density versus scan rate are shown in c) for the GC electrode and d) for the PIn-modified GC electrode. The slope of the linear fit corresponds to the double-layer capacitance, yielding a specific capacitance.

5. **Reaction kinetic vs. Mass Transport control of GC and PIn-modified GC Electrodes under Acidic Conditions**

The scan-rate dependence of the redox response of the bare GC and PIn-modified glassy carbon electrode were analyzed to support the assumption of a diffusion-controlled process. Anodic and cathodic peak currents extracted from cyclic voltammograms at different scan rates were plotted as a function of the square root of the scan rate (Figure S6). The resulting linear relationships are consistent with a reaction diffusion-controlled redox process within the PIn layer

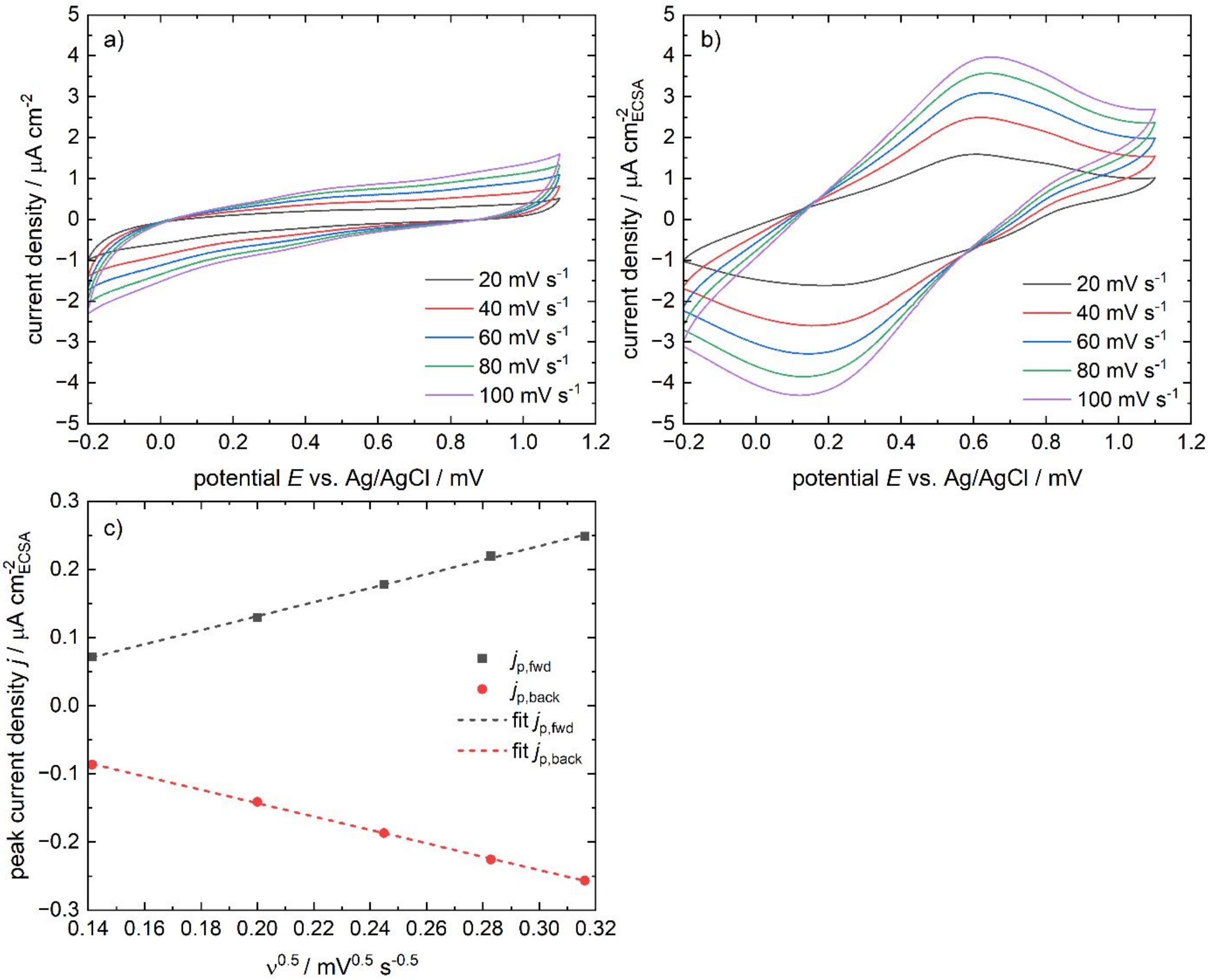

Figure S5. Cyclic voltammograms of bare GC and PIn-modified glassy GC measured in 1.0 M H_2SO_4 at scan rates between 10 and 150 mV s^{-1} (a, b), and corresponding plot for PIn-modified GC electrodes of anodic and cathodic peak currents versus the square root of the scan rate (c).

6. Identification of the Potential Window for Chronoamperometric Detection of UA on PIn-modified GC electrodes

The applied potential for chronoamperometric detection of uric acid on the PIn-modified glassy carbon electrode was optimized by comparing the current responses at different constant potentials in 0.1 M PBS (pH 7.0). As shown in Figure S6, the signal amplitude and signal-to-background ratio improve with increasing potential from 0.45 to 0.5 V vs. Ag/AgCl. Based on this comparison, 0.5 V vs. Ag/AgCl was selected as the operating potential for all chronoamperometric uric acid measurements.

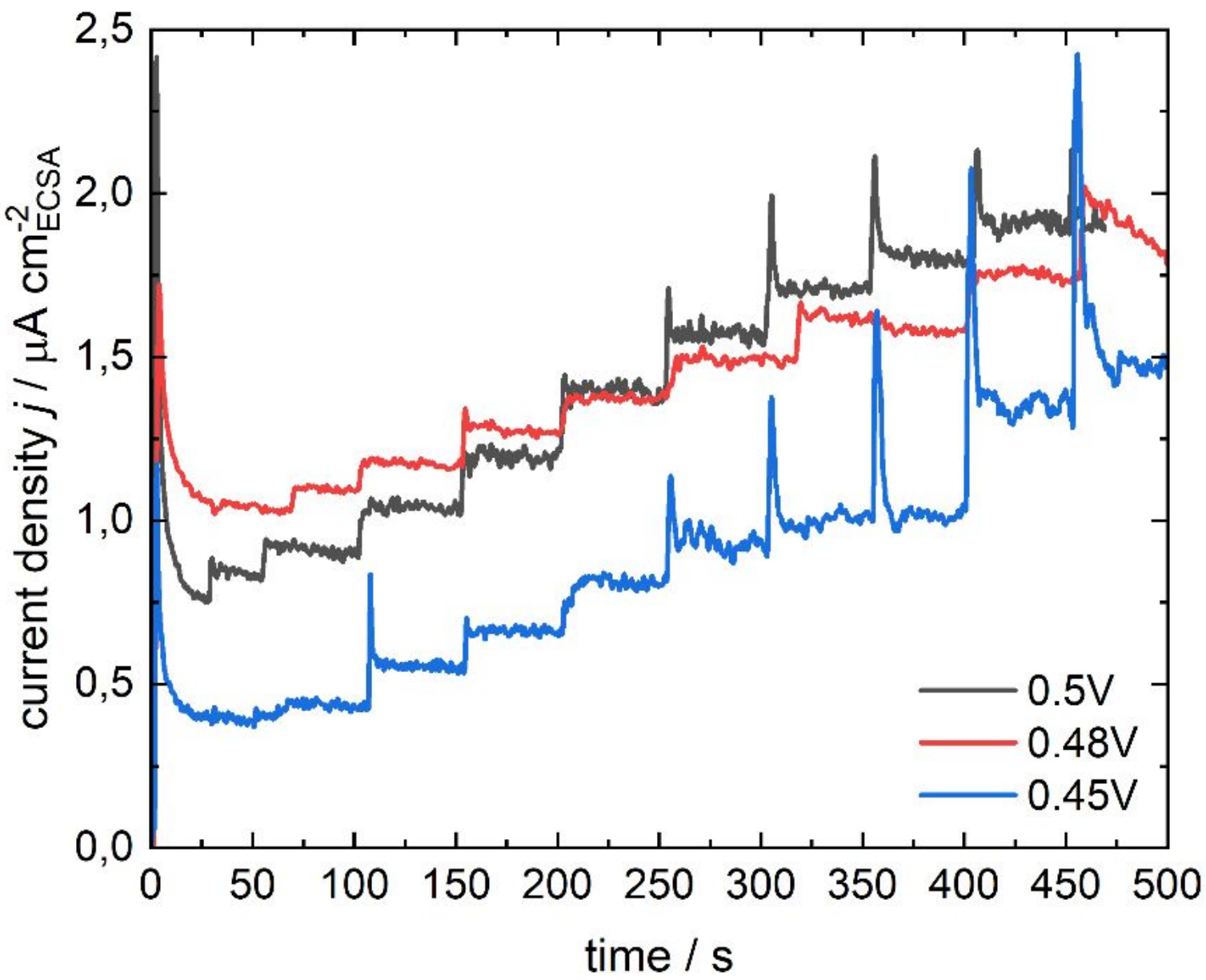

Figure S6. Comparison of the chronoamperometric response of the PIn-modified glassy carbon electrode toward uric acid in 0.1 M PBS (pH 7.0) at applied potentials of 0.45, 0.48, and 0.50 V vs. Ag/AgCl.

7. Comparison of the analytical performance of the PIn-modified glassy carbon electrode with previously reported uric acid sensors

The analytical performance of the PIn-modified glassy carbon electrode is compared with previously reported uric acid sensors based on conjugated polymers in Table S2.

Table S2 Performance comparison between PIn-coated electrodes and previously published literature of conducting polymers.

Electrode material[a]	pH	Linearity / µM	Sensitivity / $\mu A\ \mu M^{-1}\ cm^{-2}$	LOD / $\mu mol\ L^{-1}$	Reference
Poly(3,4 ethylenedioxythiophene) (PEDOT)	6.6	6-100	2.5	7.0	[1]
Poly(glyoxal-bis(2-hydroxyanil))	5.0	1.0-200	1.292	0.3	[2]
Over-oxidized Poly(*p*-aminophenol)	7.4	0.1-40.0	1.40	0.18	[3]
Poly(tartrazine)	7.5	0.34-60 and 70-140	-	0.10	[4]
Poly(proline)	6.5	100-200	-	4.7	[5]
ErGO/PEDOT:PSS modified GC	6.8	10-100	2.21	1.08	[6]
PIn/GC	7.0	10-100	14.75	12	This work

[a] all experiments were done in phosphate buffer solution (PBS) as electrolyte.

8. Reusability of PIn-modified GC Electrodes for UA Detection

The reusability of the PIn-modified GC electrode for uric acid detection was evaluated by five consecutive measurements with the same electrode (Figure S8); the corresponding current densities are summarized in Table S3.

Table S3. Reusability test of UA detection on PIn-modified GC-electrodes.

Number of repeated experiments	S1	S2	S3	S4	S5
Current density / $\mu A\ cm_{ECSA}^{-2}$	0.507	0.493	0.490	0.488	0.479

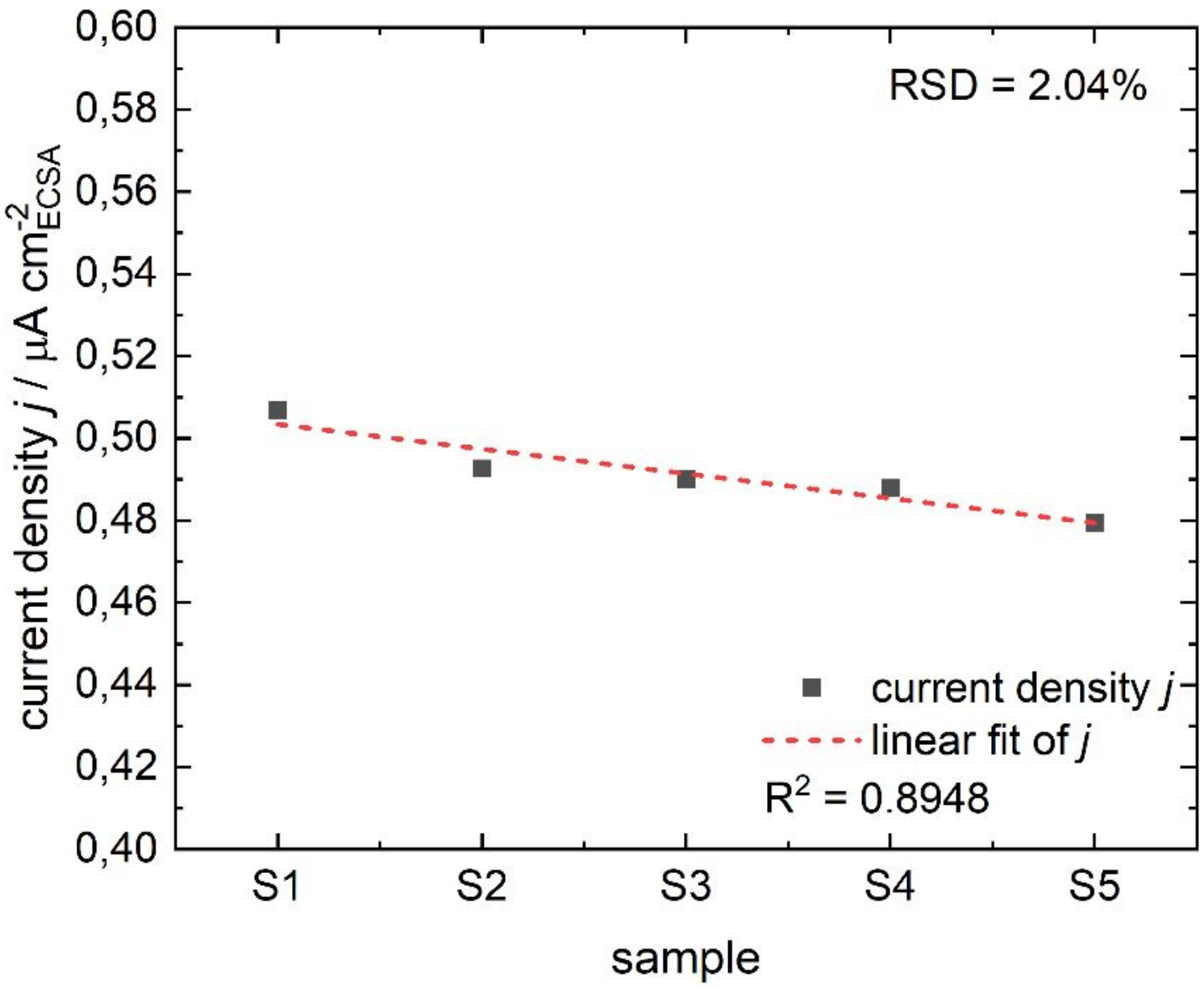

Figure S7. Reusability test of UA dectection on PIn/GC-modified electrodes. The relative standard deviation (RSD) is calculated for 5 consecutive measurements of the same electrode

9. Reproducibility of PIn-modified GC Electrodes for UA Detection

The reproducibility of the PIn-modified GC electrodes for UA detection was assessed by measuring CVs of uric acid with five independently electrodes (Figure S9); the corresponding current densities and statistical parameters are listed in Table S4 and Figure S10.

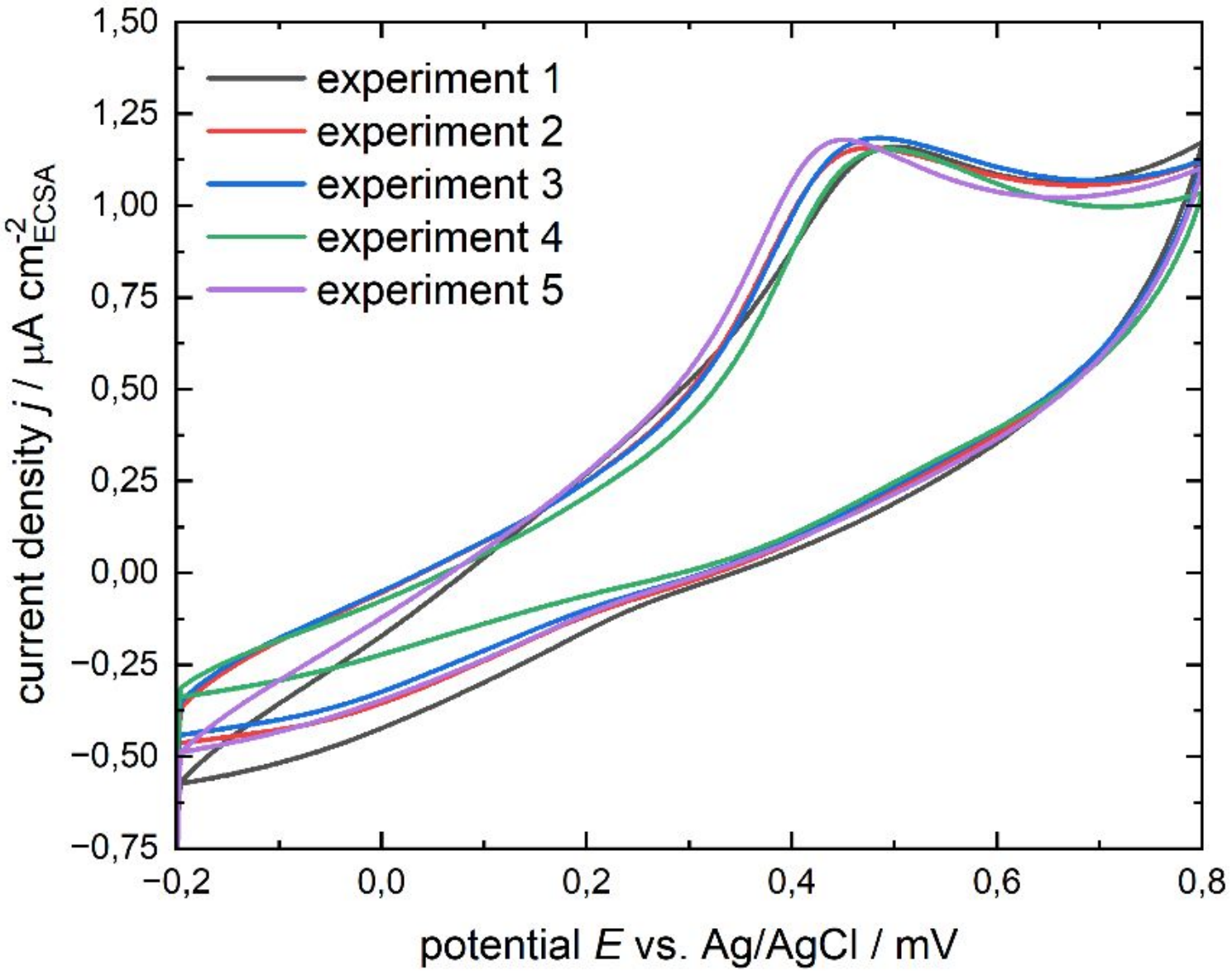

Figure S8. CVs of 5 independent measurements of PIn/GC-modified Electrodes during UA sensing.

Table S4. Reproducibility test of UA detection on PIn/GC-modified electrodes.

Number of repeated experiment	E1	E2	E3	E4	E5	mean value / $\mu A\ cm_{ECSA}^{-2}$	RDS / %
Current density / $\mu A\ cm_{ECSA}^{-2}$	1.159	1.157	1.184	1.153	1.179	1,167	1.01 %

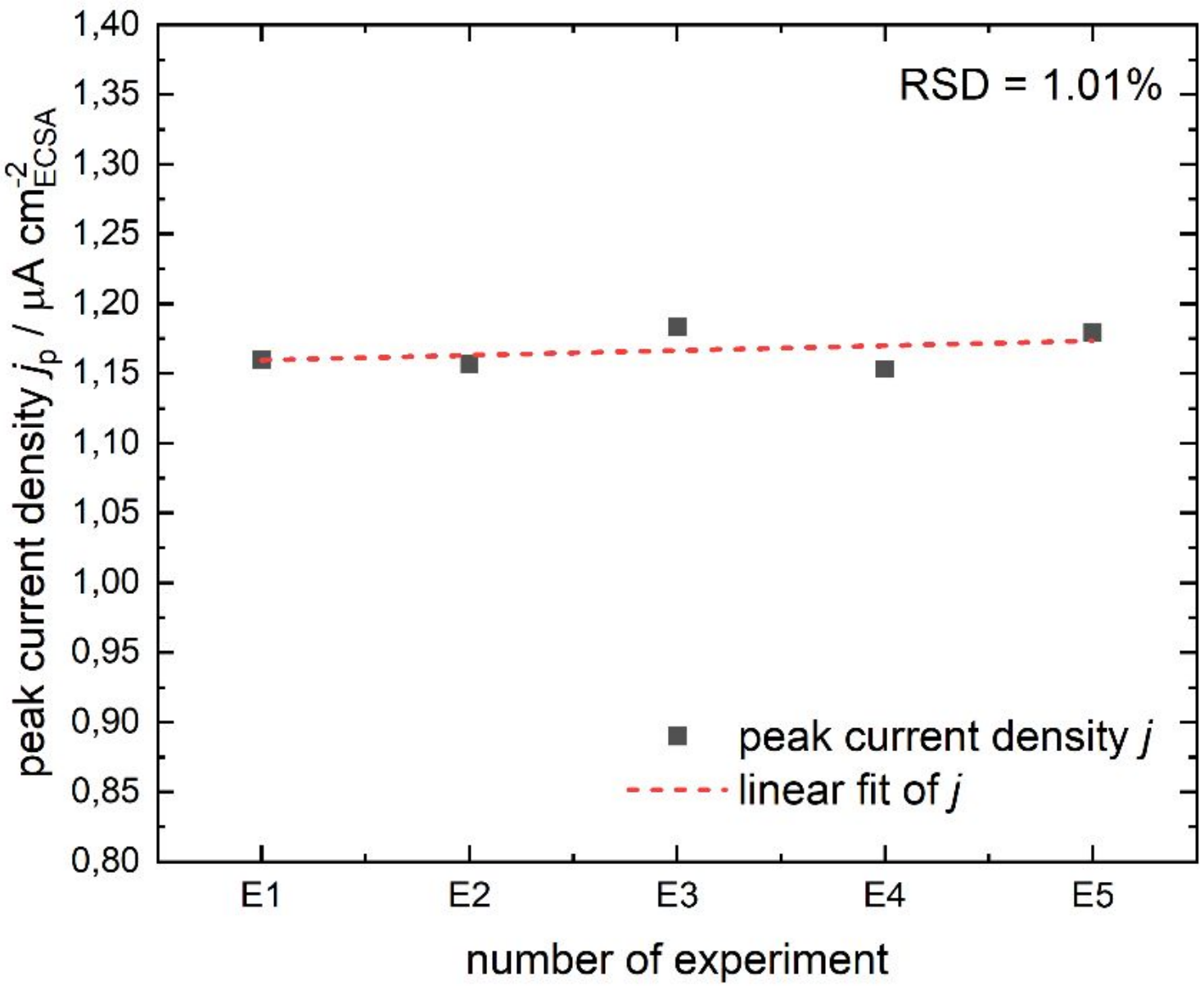

Figure S9. Reproducibility of the peak current densities j_p on PIn/GC-modified electrodes during UA sensing. The relative standard deviation (RSD) is calculated for 5 independent measurements.

10. Short-term Stability of PIn-modified GC Electrodes

The short-term stability of the PIn-modified GC electrode under chronoamperometric operation was evaluated by monitoring the current response toward uric acid over 1200 s; the corresponding stability profile is shown in Figure S10.

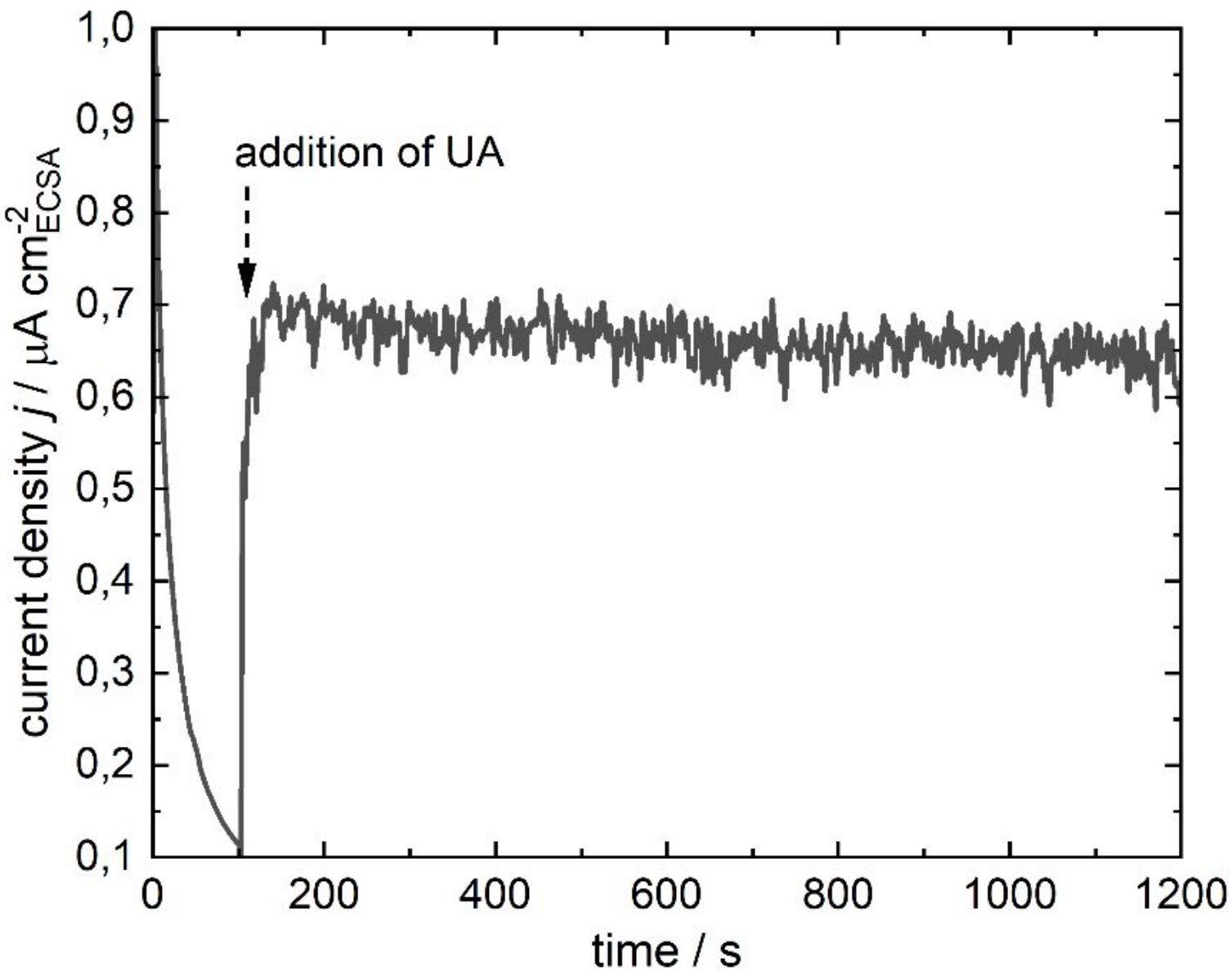

Figure S10. Stability of PIn/GC-modified electrode during UA sensing at 0.5 V vs. Ag/AgCl for 1200 s.

S11. Selectivity toward a Multicomponent Interferent Mixture

To further evaluate the anti-interference capability of the PIn-modified GC electrode, the DPV response of 0.30 mM uric acid (UA) was compared in the absence and presence of all interfering species simultaneously. A mixed solution containing 0.30 mM UA together with 0.10 mM ascorbic acid (AA), 0.001 mM dopamine (DA), 5 mM glucose, and NaCl at physiological level was prepared in 0.1 M PBS (pH 7.0), and its DPV response was recorded under identical conditions. The UA oxidation peak (≈ 0.44 V) remained essentially unchanged relative to UA alone, corresponding to a total interference of only 1.3% (Figure S10a and b). This confirms that the simultaneous presence of these physiologically relevant species has a negligible effect on the UA signal and demonstrates the high selectivity of the sensor in complex matrices.

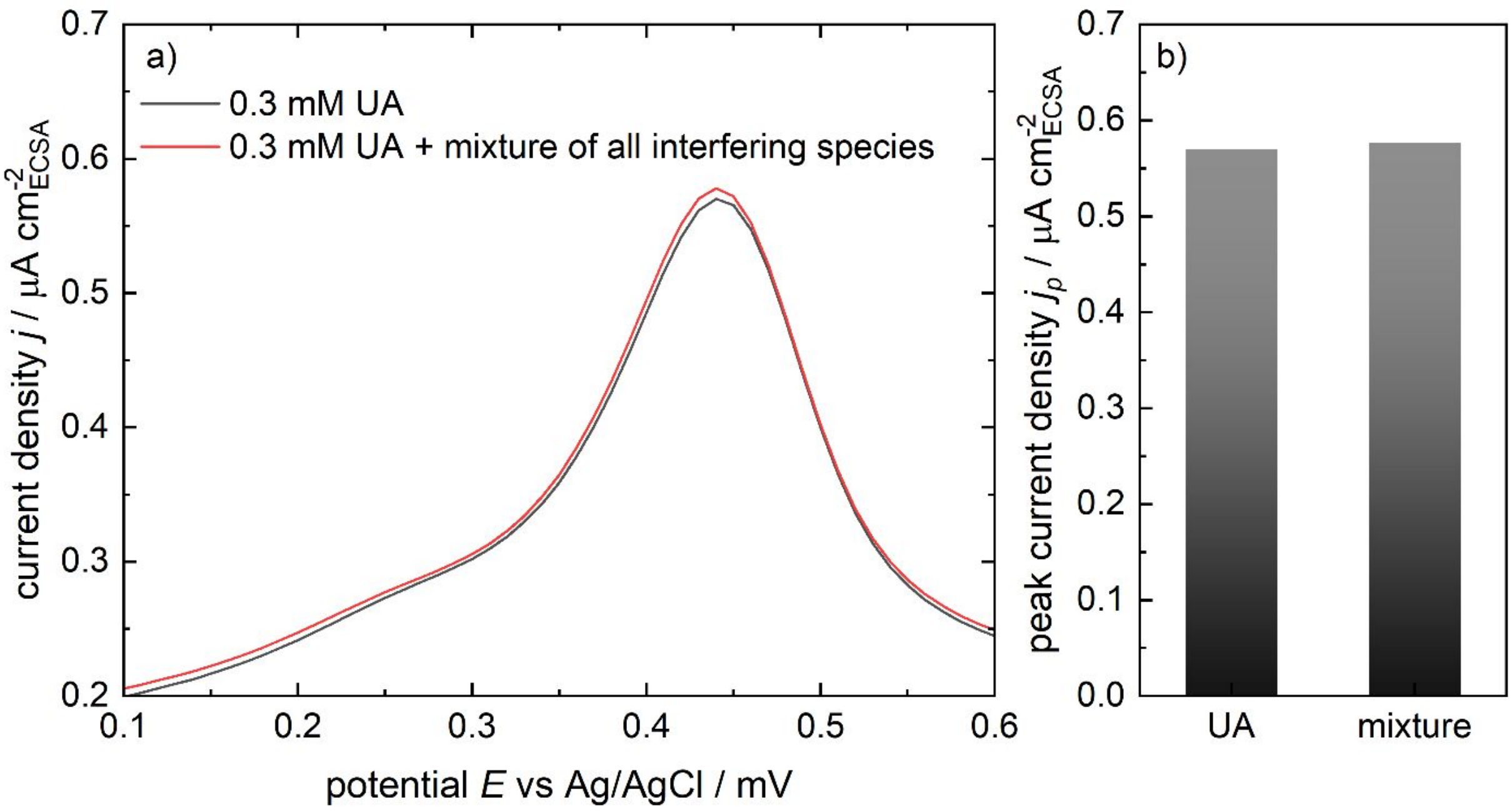

Figure S11. a) DPV response of 0.30 mM UA alone and of a multicomponent mixture (0.30 mM UA + 0.10 mM AA + 0.001 mM DA + 5 mM glucose + NaCl at physiological level) in 0.1 M PBS (pH 7.0). b) Figure Sx2. Comparison of the UA anodic peak current for 0.30 mM UA alone versus the multicomponent mixture, corresponding to a total interference of ≈ 1.3 %.

S11. Blood Serum Experiments

Blood samples were collected from seven patients with institutional approval and informed consent, centrifuged to separate the serum, and diluted ten-fold with 0.1 M PBS (pH 7.0). Endogenous uric acid was quantified by the chronoamperometric standard-addition method at 0.5 V vs. Ag/AgCl using the PIn-modified GC electrode: for each sample, the steady-state current was recorded after successive additions of standard UA solution, and the endogenous concentration was obtained from the intercept of the resulting current–concentration plot. All measurements were performed in triplicate. Figure S11 Shows a representative series of blood serum experiments.

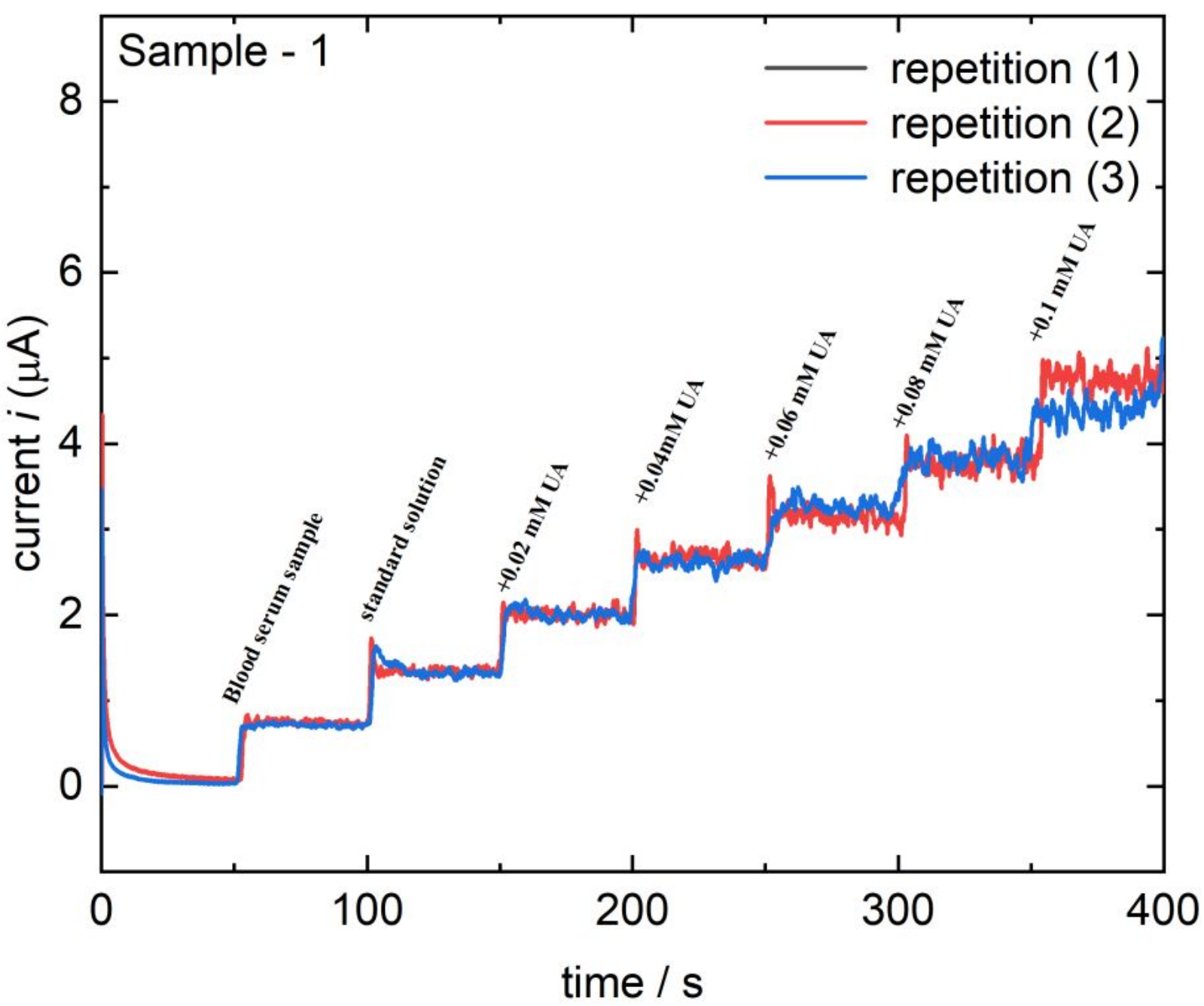

Figure S12. Chronoamperometric standard-addition plots for the determination of uric acid in diluted human blood serum (0.1 M PBS, pH 7.0; 0.5 V vs. Ag/AgCl) for a representative serum sample.

11. References

[1] M. Motshakeri, J. Travas-Sejdic, A. R. Phillips, P. A. Kilmartin, Rapid electroanalysis of uric acid and ascorbic acid using a poly (3, 4-ethylenedioxythiophene)-modified sensor with application to milk. *Electrochimica Acta*. (2018) *265*, 184-193. https://www.sciencedirect.com/science/article/pii/S0013468618301944.

[2] E. Ergün, Ş. Kart, D. K. Zeybek, B. Zeybek, Simultaneous electrochemical determination of ascorbic acid and uric acid using poly (glyoxal-bis (2-hydroxyanil)) modified glassy carbon electrode. *Sensors and Actuators B: Chemical*. (2016) *224*, 55-64. https://www.sciencedirect.com/science/article/pii/S0925400515305025.

[3] Ç. C. Koçak, Z. Dursun, Simultaneous determination of ascorbic acid, epinephrine and uric acid at over-oxidized poly (p-aminophenol) film modified electrode. *Journal of electroanalytical chemistry*. (2013) *694*, 94-103. https://www.sciencedirect.com/science/article/pii/S1572665713000817.

[4] L. Liv, M. Portakal, M. S. Çukur, B. Topaçlı, B. Uzun, Electrocatalytic determination of uric acid with the poly (Tartrazine)-Modified pencil graphite electrode in human serum and artificial urine. *ACS omega*. (2023). *8* (38), 34420-34430. https://pubs.acs.org/doi/abs/10.1021/acsomega.3c02561.

[5] E. S. D'Souza, J. G. Manjunath, C. Raril, Electrochemical determination of dopamine and uric acid using poly (proline) modified carbon paste electrode: a cyclic voltammetric study. (2021). https://ena.lpnu.ua/items/c19ea836-b478-414b-9806-7b242f650067.

[6] B. R. Putra, U. Nisa, R. Heryanto, E. Rohaeti, M. Khalil, A. Izzataddini, W. T. Wahyuni, A facile electrochemical sensor based on a composite of electrochemically reduced graphene oxide and a PEDOT: PSS modified glassy carbon electrode for uric acid detection. *Analytical Sciences*. (2022) *38* (1), 157-166. https://link.springer.com/article/10.2116/analsci.21P214.
